# The transcription factor MYB156 controls the polar stiffening of guard cell walls in poplar

**DOI:** 10.1093/plcell/koad198

**Published:** 2023-07-12

**Authors:** Lin Zheng, Yajuan Chen, Liping Ding, Ying Zhou, Shanshan Xue, Biying Li, Jianhua Wei, Hongzhi Wang

**Affiliations:** Beijing Key Laboratory of Agricultural Genetic Resources and Biotechnology, Institute of Biotechnology, Beijing Academy of Agriculture and Forestry Sciences, Beijing 100097, China; Beijing Key Laboratory of Agricultural Genetic Resources and Biotechnology, Institute of Biotechnology, Beijing Academy of Agriculture and Forestry Sciences, Beijing 100097, China; Beijing Key Laboratory of Agricultural Genetic Resources and Biotechnology, Institute of Biotechnology, Beijing Academy of Agriculture and Forestry Sciences, Beijing 100097, China; Beijing Key Laboratory of Agricultural Genetic Resources and Biotechnology, Institute of Biotechnology, Beijing Academy of Agriculture and Forestry Sciences, Beijing 100097, China; Beijing Key Laboratory of Agricultural Genetic Resources and Biotechnology, Institute of Biotechnology, Beijing Academy of Agriculture and Forestry Sciences, Beijing 100097, China; Beijing Key Laboratory of Agricultural Genetic Resources and Biotechnology, Institute of Biotechnology, Beijing Academy of Agriculture and Forestry Sciences, Beijing 100097, China; Beijing Key Laboratory of Agricultural Genetic Resources and Biotechnology, Institute of Biotechnology, Beijing Academy of Agriculture and Forestry Sciences, Beijing 100097, China; Beijing Key Laboratory of Agricultural Genetic Resources and Biotechnology, Institute of Biotechnology, Beijing Academy of Agriculture and Forestry Sciences, Beijing 100097, China

## Abstract

The mechanical properties of guard cells have major effects on stomatal functioning. Reinforced stiffness in the stomatal polar regions was recently proposed to play an important role in stomatal function, but the underlying molecular mechanisms remain elusive. Here, we used genetic and biochemical approaches in poplar (*Populus* spp.) to show that the transcription factor MYB156 controls pectic homogalacturonan–based polar stiffening through the downregulation of the gene encoding pectin methylesterase 6 (PME6). Loss of *MYB156* increased the polar stiffness of stomata, thereby enhancing stomatal dynamics and response speed to various stimuli. In contrast, overexpression of *MYB156* resulted in decreased polar stiffness and impaired stomatal dynamics, accompanied by smaller leaves. Polar stiffening functions in guard cell dynamics in response to changing environmental conditions by maintaining normal stomatal morphology during stomatal movement. Our study revealed the structure–function relationship of the cell wall of guard cells in stomatal dynamics, providing an important means for improving the stomatal performance and drought tolerance of plants.

## Teaser

Polar stiffening of the guard cell mediated by MYB156 is essential for stomatal dynamics, and manipulating this mechanical property provides a promising approach for engineering stomatal function.

**IN A NUTSHELL**

**Background:** Stomata, tiny pores on plant leaves, control gas exchange between plants and their environment. The functioning of stomata relies on the properties of guard cell walls. Traditional thinking suggests that the difference in thickness between inner and outer cell walls is crucial, but a recent idea highlights the importance of reinforced stiffness in the polar regions of the guard cell walls. However, the genes responsible for these wall properties are unknown. Thus, the mechanics of stomatal movement remain a mystery.

**Question:** What property of guard cell walls is vital for proper functioning, and can we manipulate it through genetic engineering?

**Findings:** The stiffness of guard cell walls, particularly in polar regions, is crucial for stomatal movement. An important player in this process is the MYB156 transcription factor in *Populus*. By regulating the activity of pectin methylesterase 6 (PME6), MYB156 influences the amount of de-esterified pectin in the polar regions of guard cell walls, thereby affecting their stiffness. Loss of *MYB156* leads to increased polar stiffness, causing stomata to respond more swiftly to various stimuli. Conversely, excessive expression of *MYB156* decreases polar stiffness and impairs stomatal movement. The polar stiffening of guard cell walls plays a significant role in responding to environmental changes. This study sheds light on the mechanical properties of guard cell walls during stomatal movement, with the potential to enhance plant drought tolerance by engineering this specific property.

**Next steps:** Our research suggests that by enhancing polar stiffening, we can improve the performance of guard cells and plant drought tolerance. Genetic engineering techniques can be employed to target the *MYB156* gene and enhance the function of guard cells. Additionally, further investigation into how polar stiffening is affected in mutated guard cells can reveal additional players involved in this process.

## Introduction

Stomata, a key innovation during plant evolution, control gas exchange between the plant and the environment (Munemasa et al. 2015; Shtein et al. 2017). Since land plants lose over 95% of their water via transpiration through stomatal pores, engineering of stomatal behavior represents a valuable tool for designing new crops with higher stress tolerance and productivity through improving stomatal response speed (Lawson and Blatt 2014; Lawson and Vialet-Chabrand 2019).

Two main factors, namely, osmotic adjustment and cell wall–related stomatal structure, determine stomatal behavior (Lawson and Vialet-Chabrand 2019). The molecular mechanism underlying the adjustment of osmotic pressure in the guard cells has been well established (Kim et al. 2010; Munemasa et al. 2015; Assmann and Jegla 2016). Furthermore, the guard cell wall controls stomatal response speed and the degree of pore opening/closure through imposing mechanical constraints on the deformed cells, which are central to stomatal functioning (Woolfenden et al. 2017, 2018; Lawson and Vialet-Chabrand 2019). However, our understanding of how cell wall mechanics function in stomatal movement remains limited.

Recent work identified an additional component of guard cell mechanics that appears to be significant for stomatal opening, namely, increased stiffness of the cell wall in the stomatal polar regions, where the end of 2 guard cell pairs meets (Carter et al. 2017; Dow 2017), challenging the traditional model of differential thickening of walls (Esau 1967; DeMichele and Sharpe 1973). Although the roles of polar stiffening in guard cell functioning have been shown by computational modeling and functional stomata assays, the genetic basis and key molecular players are still unclear. Revealing this information will help enable efforts to improve stomatal performance through the genetic modification of polar stiffening.

It has been proposed that polar stiffening involves mechanical pinning down of the guard cell ends by pectic homogalacturonan (HG), which restricts the increase of stomatal complex length and therefore increases the stomatal width during opening. As an important component of the primary cell wall, pectin regulates the mechanical properties of cell walls and chemical alterations of pectin underlie changes in cell wall rheology, softening, and stiffening (Levesque-Tremblay et al. 2015). Chemical modifications of HG involve methylesterification and polymerization, which are controlled by pectin methylesterases (PMEs), polygalacturonases (PGs), and pectate lyase-like proteins (PLLs) (Senechal et al. 2014). The demethylesterification of HG by PMEs leads to increased or decreased cell wall stiffness depending on whether the demethylesterified HG is crosslinked with Ca^2+^ or degraded by PGs and/or PLLs (Levesque-Tremblay et al. 2015).

In the polar regions of guard cells, the enrichment of demethylesterified HG polymers is essential for polar stiffening, as revealed by PG treatment (Carter et al. 2017). Genetic manipulation of HG-modifying enzymes, including PMEs (Amsbury et al. 2016), PGs (Rui et al. 2017), and PLLs (Chen et al. 2021), alters the degrees of methylesterification and polymerization of HG and the abundance of calcium-crosslinked HG in the guard cell walls, thereby disrupting proper stomatal functioning. Little is currently known about how this pectic HG-based polar stiffening is regulated in the guard cells. We hypothesize that transcriptional regulators and signaling cascades might participate in the regulation of stomatal polar stiffening.

Poplar (*Populus* spp.) is a model tree with broad leaves and considerable water loss via the stomata. By integrating genetic and biochemical approaches, herein, we report that stomatal polar stiffening in *Populus* is controlled by MYB156-mediated downregulation of *pectin methylesterase 6* (*PME6*) and the associated altered HG methylesterification in the polar regions of guard cells.

## Results

### MYB156 functions in stomatal dynamics

**Figure 1.**
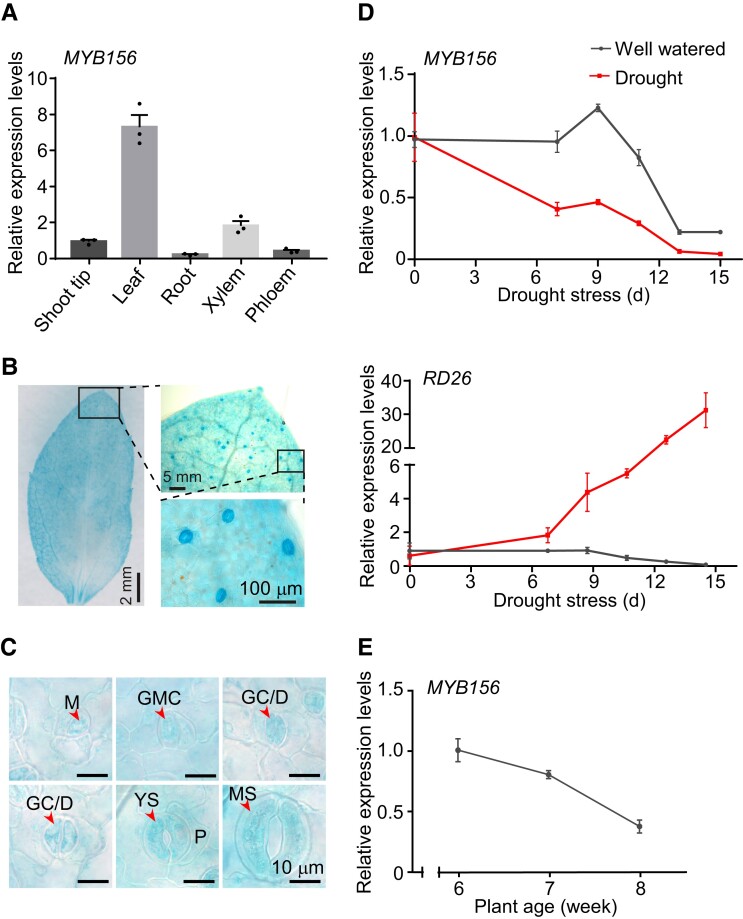
Expression Analysis of *MYB156*. **A)** The expression pattern of *MYB156* in various tissues by reverse transcription quantitative PCR (RT-qPCR). The data are presented as means ± Se for three plants. Black dots represent the individual data for each plant replicate. **B)***MYB156* promoter-driven *b*-glucuronidase (*GUS*) expression in transgenic *Populus* leaves, with boxes highlighting the regions enlarged in the corresponding images. Bar = 100 *μ*m. **C)***MYB156* promoter-driven *GUS* expression in pavement cells, and various developmental stages of stomata. The representative images are shown. M, meristemoid (triangular); GMC, guard mother cell (oval); GC/D, guard cell undergoing development; YS: young stomata (with a length approximately equal to its width); MS, mature stomata (with a larger length compared to its width); P, pavement cell. Bar = 10 *μ*m. **D)** The expression of *MYB156* under drought stress by RT-qPCR. The drought-inducible *RD26* gene was used as a positive control for drought treatment. Values are means ± Se for eight plants. **E)** The expression of *MYB156* in mature LPI6 leaves (LPI, Leaf Plastochron Index, which was used as an indicator of the leaf age, with LPI6 representing the sixth leaf from the plant top) from plants of different ages using RT-qPCR. The data are presented as means ± Se for three plants. *Populus ACTIN2* was used as an internal control for RT-qPCR analysis in **A)**, **D)**, and **E)**.

The *Populus* transcription factor MYB156 is known to modulate secondary cell wall biosynthesis (Yang et al. 2017; Zheng et al. 2019). In our functional study of MYB156, we found that *MYB156* was predominantly expressed in leaf tissue (Fig. 1A), including pavement cells and various developmental stages of stomata (Fig. 1, B and C). In mature leaves, the expression level of *MYB156* was substantially higher in guard cells than in the surrounding pavement cells (Fig. 1B). To explore the physiological roles of MYB156 in plants, we monitored changes in *MYB156* expression in *Populus* plants undergoing drought stress using reverse transcription quantitative PCR (RT-qPCR). Our pilot experiments suggested that 11 d of drought treatment would induce a wilted phenotype in 2-mo-old *Populus* plants. We sampled leaf tissues for RT-qPCR analysis at 0, 7, 9, 11, 13, and 15 d after water deprivation in the subsequent drought treatment experiments, using well-watered plants as controls.

While the expression of the drought-inducible RD26 gene was dramatically induced under drought treatment, the expression of *MYB156* greatly decreased during the first 9 d of drought treatment (Fig. 1D) compared to the well-watered control plants, suggesting that *MYB156* is a drought-responsive gene. We also observed decreased expression of *MYB156* after day 9 in well-watered control plants, implying age-dependent expression of *MYB156*. This was supported by a further RT-qPCR analysis of *MYB156* expression in leaves at LPI6, where leaf plastochron index (LPI) was used as an indicator of the leaf age, with LPI6 representing the 6th leaf from the plant top (Larson and Isebrands 1971). In LPI6 leaves from 6-, 7-, and 8-wk-old plants, *MYB156* expression decreased dramatically as the plants grew (Fig. 1E), suggesting that *MYB156* expression is regulated by developmental cues. Taken together, these data demonstrate that *MYB156* is predominantly expressed in leaf tissue and guard cells and responds to drought stress, suggesting it may have a role in stomatal functioning.

To investigate the role of MYB156 in stomatal function, we generated knock-out mutants via CRISPR/Cas9 genome-editing technology in *Populus davidiana* × *Populus bolleana* (Shanxin Yang), a heterozygous diploid. Two different *MYB156*-gRNAs were designed from the reference coding sequence of the first exon of *MYB156*, where no single-nucleotide polymorphisms (SNPs) are present between the 2 *MYB156* alleles. This ensures that both alleles will be targeted by the gRNAs. Out of hundreds of transgenic plants, we identified 2 biallelic mutants (Supplemental Fig. S1, A to D). Each mutant was derived from an individual gRNA targeting. In the first mutant (*myb156 #1*), 1 allele of *MYB156* (*myb156-1*) has a 1-bp deletion, leading to translational frameshift, and the other allele (*myb156-2*) has a 21-bp insertion leading to premature termination. In the other mutant (*myb156 #2*), there is a 1-bp deletion in 1 allele (*myb156-3*) and 1-bp insertion in the other allele (*myb156-4*), both leading to a translational frameshift. These 2 mutants were used for further analysis in this study.

Stomatal response to various stimuli was assayed in the *myb156* mutants. To this end, light was applied to the excised leaves to induce stomatal opening. The phytohormone abscisic acid (ABA) and the osmoticum mannitol were used to induce stomatal closing. Shifts in CO_2_ conditions and drought stress were applied to whole plants to monitor the real-time physiological outcome of stomatal movement. Along with measuring stomatal pore width, stomatal conductance is sometimes used to interpret the degree of stomatal opening and closure. This is done when the plants have comparable physiological states, are grown under the same environmental conditions, and have similar stomatal density.

**Figure 2.**
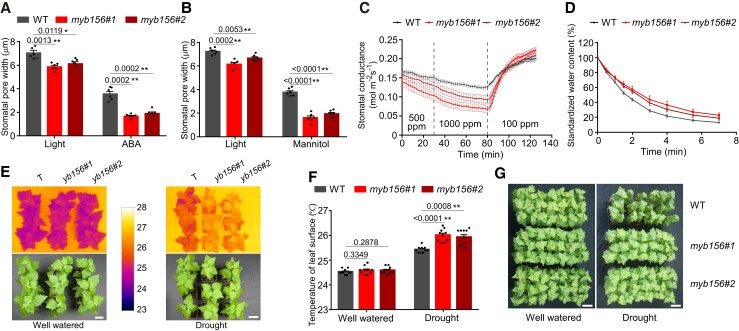
Loss of *MYB156* Enhances Stomatal Dynamics and Response Speed. **A)** Stomatal response to light and abscisic acid (ABA) treatment in wild-type (WT) and *myb156* mutants. Stomatal opening was induced under light for 2 h, following which stomatal closure was induced by 20 mm ABA for 3 h. The data are presented as means ± Se for six plants per genotype. Each plant was measured for the mean pore width of 20 stomata, which were randomly sampled from two fully expanded LPI6 and 7 leaves (LPI, Leaf Plastochron Index, which was used as an indicator of the leaf age, with LPI6 and 7 respectively representing the sixth and seventh leaf from the plant top). Black dots represent the individual data for each plant replicate. **B)** As in **(A)** but treated with 1 M mannitol for 3 h to induce stomatal closure. **C)** Stomatal conductance response to changing CO_2_ concentration in WT and *myb156* mutants. The time-course of stomatal conductance was examined under the indicated CO_2_ condition for a period of time. The data are presented as means ± Se of 10 plants per genotype. Note the larger dynamic range and more rapid response to changing CO_2_ concentration in *myb156* mutant stomata. **D)** Water loss in WT and *myb156* mutants. Time-course of cumulative water loss from the leaves via transpiration was examined at the indicated time points after the leaves were detached. The data are presented as means ± Se of 10 plants per genotype. Note the slowed-down water loss rate in the detached leaves in the *myb156* mutants. **E)** The increased leaf surface temperature in *myb156* under drought stress. Thermal images were taken at 9 days after withholding water for drought stress, with well-watered plants as the control. Representative photographs are shown. Bar = 10 cm. **F)** Quantification of the thermal image data in **(E)**. The data are presented as means ± Se of 10 plants per genotype per treatment. Black dots represent the individual data for each plant replicate. **G)** Drought tolerance of *myb156* mutants. Plants were grown for two months and then exposed to dehydration by withholding water, with well-watered plants using as the control. The *myb156* mutants showed a phenotype of tolerance after drought treatment for 11 days. For each genotype, 21 plants were grown. The experiments were conducted three times, with similar results obtained each time. Bar = 10 cm. Asterisks in **(A)**, **(B)**, and **(F)** represent significant differences (*P < 0.05; **P < 0.01) compared with WT, as determined by Student's t test. The precise *P* values are also provided.

The *myb156* mutant stomata closed more tightly in response to ABA, mannitol, and elevated CO_2_ than the wild-type (WT) stomata (Fig. 2, A to C) and opened as widely as the WT stomata under depleted CO_2_ (Fig. 2C). Furthermore, *myb156* stomata responded more rapidly to shifts in CO_2_ conditions than WT stomata. When the CO_2_ concentration was elevated, stomatal conductance (*g_s_*) dropped more rapidly in *myb156* than in WT; when CO_2_ was depleted, *g_s_* increased more rapidly in *myb156* (Fig. 2C). These data indicate that the loss of *MYB156* expression improves the stomatal dynamic range and response speed.

Consistent with the faster closing of *myb156* stomata, the water loss rates in *myb156* mutant leaves were slower than those in WT leaves (Fig. 2D). Although the leaf surface temperature of the *myb156* mutant did not differ from that of WT plants under well-watered conditions, after withholding water for 9 d, the temperatures increased compared with WT (Fig. 2, E and F), suggesting that the stomata of *myb156* experienced accelerated closing during drought stress. As a result, *myb156* plants showed a drought-tolerant phenotype (Fig. 2G). Overall, the data in Figs. 1 and 2 reveal the physiological functions of MYB156 in stomatal dynamics.

**Figure 3.**
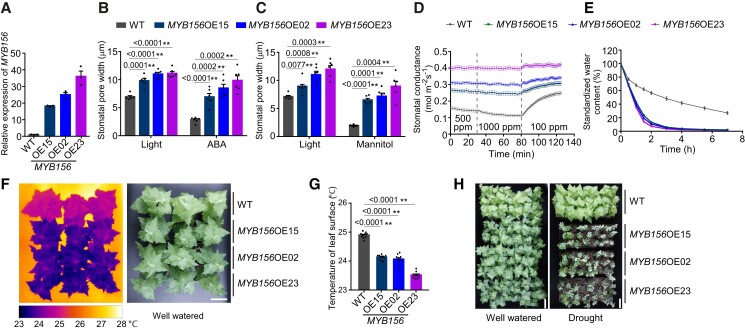
*MYB156* Overexpression Impairs Stomatal Dynamics. **A)***MYB156* expression in *MYB156*-overexpressing *Populus* by reverse transcription quantitative PCR (RT-qPCR). The *Populus ACTIN2* gene was used as an internal control, and error bars represent the Se of three biological replicates, where each replicate consisted of an independent RNA pool. Each RNA pool was obtained by pooling RNA samples from three individual plants. Black dots represent the individual data for each biological replicates. **B)** Stomatal response to light and abscisic acid (ABA) treatment in wild-type (WT) and *MYB156* overexpression (OE) plants. Stomatal opening was induced under light for 2 h, following which stomatal closure was induced by 20 mm ABA for 4 h. The data are presented as means ± Se for six plants per genotype. Each plant was measured for the mean pore width of 20 stomata, which were randomly sampled from two fully expanded LPI6 and 7 leaves (LPI, Leaf Plastochron Index, which was used as an indicator of the leaf age, with LPI6 and 7 respectively representing the sixth and seventh leaf from the plant top). Black dots represent the individual data for each plant replicate. **C)** As in **(B)** but treated with 1 M mannitol for 4 h to induce stomatal closure. **D)** Stomatal conductance response to changing CO_2_ concentration in WT and *MYB156*OEs. The time-course of stomatal conductance was examined under the indicated CO_2_ condition for a period of time. The data are presented as means ± Se of 10 plants per genotype. **E)** Water loss in WT and *MYB156*OEs. Time-course of cumulative water loss from the leaves via transpiration was examined at the indicated time points after the leaves were detached. The data are presented as means ± Se of 10 plants per genotype. **F)** The cooler leaf surface in *MYB156*OEs under well-watered conditions. Representative photographs are shown. Bar = 10 cm. **G**) Quantification of the thermal image data in **(F)**. The data are presented as means ± Se of 10 plants per genotype. Black dots represent the individual data for each plant replicate. **H)** Drought tolerance of WT and *MYB156*OEs. Plants were grown for two months and then exposed to dehydration by withholding water, with well-watered plants using as the control. The *MYB156*OEs showed an extremely sensitive phenotype after drought treatment for 9 days. For each genotype, 21 plants were grown. The experiments were conducted three times, with similar results obtained each time. Bar = 10 cm. Asterisks in **(B)**, **(C)**, and **(G**) represent significant differences (*P < 0.05; **P < 0.01) compared with WT, as determined by Student's t test. The precise *P* values are also provided.

Meanwhile, we generated *MYB156*-overexpressing lines by constitutively overexpressing *MYB156* under the cauliflower mosaic virus *35S* promoter (*Pro35S*) in WT *Populus*. Three independent overexpression (OE) lines with 22–50 times the normal levels of *MYB156* transcript, namely, *MYB156* OE15, OE02, and OE23, were chosen for the analysis of stomatal response to various stimuli (Fig. 3A). In contrast to the stomata of *myb156* mutants, the *MYB156*OE stomata displayed a much larger aperture than those of the WT under all conditions applied here and almost did not close under ABA, mannitol, and elevated CO_2_ (Fig. 3, B to D), suggesting that *MYB156* overexpression significantly impaired stomatal dynamics. Accordingly, water loss from the detached leaves was accelerated in *MYB156*OE lines compared with the WT plants (Fig. 3E) and the leaf surface temperature was significantly decreased owing to enhanced evaporation cooling from a larger stomatal aperture (Fig. 3, F and G). As a result, *MYB156*OEs showed extreme sensitivity to drought stress (Fig. 3H). These data indicate that the *MYB156* overexpression significantly impaired stomata dynamics, leading to an extremely narrower range of stomatal opening/closure in response to various stimuli.

### PME6 functions directly downstream of the transcriptional repressor MYB156 in stomatal dynamics

**Figure 4.**
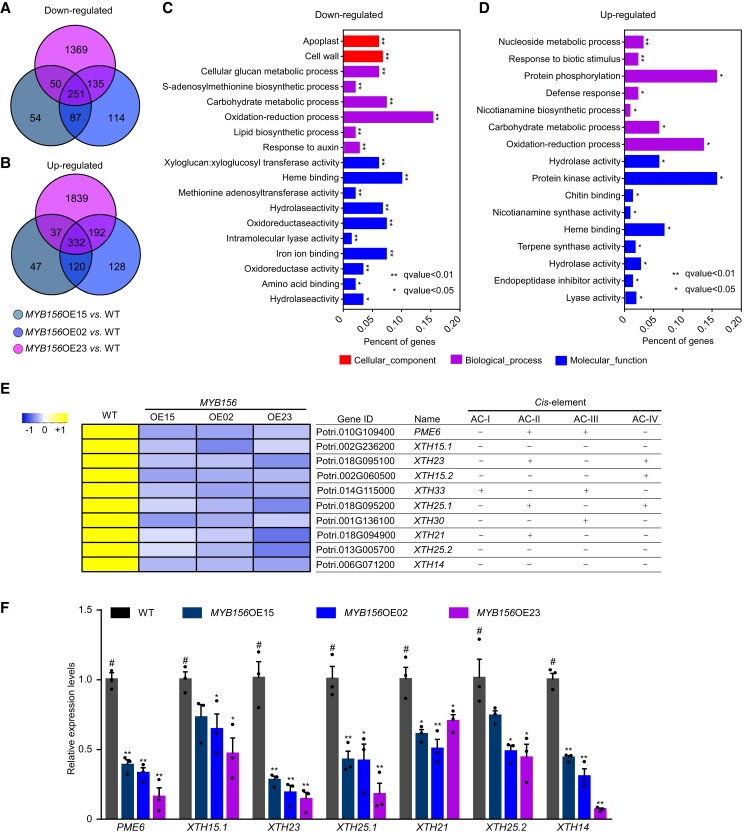
Identification of the MYB156 Target Genes. **A**, **B)** Venn diagram showing overlaps among genes downregulated and upregulated in *MYB156* overexpression (OE) lines (*MYB156*OE15, *MYB156*OE02, and *MYB156*OE23) compared with wild-type (WT). Differentially expressed genes were identified based on a two-fold cut-off value (*P* < 0.05). **C**, **D)** The Gene Ontology (GO) enrichment analysis of 251 downregulated genes and 332 upregulated genes. **E)** Hierarchical clustering analysis and *cis*-elements screening within the promoter regions of Cell Wall category genes from the GO enrichment of downregulated genes. Names of the genes are adopted from the *Populus trichocarpa* genome v. 4.1 (https://phytozome-next.jgi.doe.gov/info/Ptrichocarpa_v4_1). **F)** The analysis of gene expression in the Cell Wall category by reverse transcription quantitative PCR (RT-qPCR). The LPI6 leaves (LPI, Leaf Plastochron Index, which was used as an indicator of the leaf age, with LPI6 representing the sixth leaf from the plant top) were harvested for RNA extraction. The *Populus ACTIN2* gene was used as an internal control. The data are presented as means ± Se (n = 3 plants per genotype). Black dots represent the individual data for each plant replicate. *Significant at *P* < 0.05, **significant at *P* < 0.01 compared with WT (#, representing the specific control for each comparison) based on Student's *t*-test.

To fully understand how MYB156 functions in the stomata, transcriptome-based screening of target genes was performed in the *MYB156*-overexpressing lines. A total of 583 (Log_2_ ratio ≥ 1.0, *P* ≤ 0.01, and FDR ≤ 0.05) differentially expressed genes were found in all 3 transformation lines, including 251 downregulated genes and 332 upregulated genes (Fig. 4, A and B). Gene Ontology (GO) functional clustering analysis of differentially expressed genes did not demonstrate any gene clusters directly related to ABA signaling and ion transport, and the most interesting GO term was the “Cell Wall” component in the downregulated genes (Fig. 4, C and D), including a pectin methylesterase gene (*PME6*) and 9 xyloglucan endotransglucosylase/hydrolase genes (*XTHs*) (Fig. 4E).

The RT-qPCR analysis confirmed that the transcript levels of *PME6* and 6 *XTHs* were decreased in the *MYB156*-overexpressing lines compared with WT (Fig. 4F). The promoter regions (∼1,500 bp) of those genes in the *Populus trichocarpa* genome were screened for AC *cis*-elements (AC-I, ACCTACC; AC-II, ACCAACC; AC-III, ACCTAAC; and AC-IV, ACCAAAC) that MYB156 binds to (Zheng et al. 2019), and 7 out of 10 candidate genes were found to contain at least 1 of those elements (Fig. 4E), suggesting that they might be directly regulated by MYB156. Among those candidate genes, the expression levels of *PME6* and *XTH23* (Fig. 4F) showed substantial negative association with the expression levels of *MYB156* (Fig. 3A) in those 3 *MYB156*-overexpressing lines. Given that HG modifications have been reported to play significant roles in the mechanics and dynamics of the stomata (Amsbury et al. 2016; Huang et al. 2017; Rui et al. 2017; Chen et al. 2021), we selected *PME6* as a candidate to study the molecular mechanism of how MYB156 functions in stomatal dynamics.

**Figure 5.**
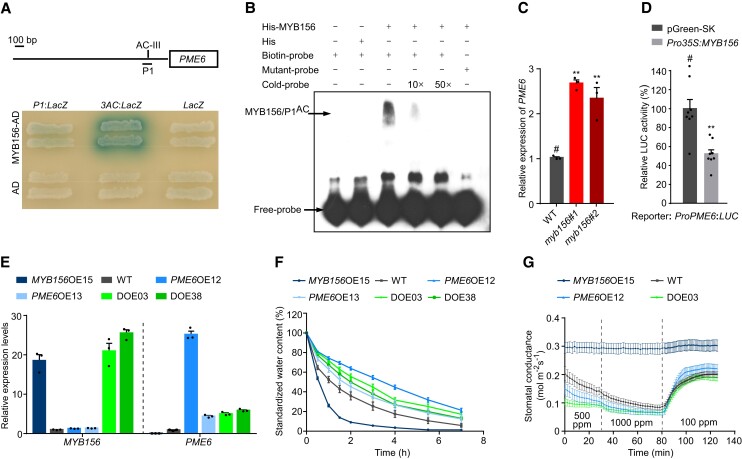
PME6 Functions Directly Downstream of the Transcriptional Repressor MYB156 in Stomatal Dynamics. **A)** Yeast one-hybrid (Y1H) assays of MYB156 binding to the promoter of *PME6*. Schematic of the *PME6* promoter is shown, and an 83-bp fragment (P1) with an AC-III (ACCTAAC) element is marked. **B)** Electrophoretic mobility shift assay showing the binding of MYB156 to the promoter of *PME6 in vitro.* His-MYB156 bound to the P1^AC^ fragment (the biotin-probe), and the binding signal disappeared when the sequence of the AC-III element was mutated into AAATAAA (the mutant-probe). The positions of hysteresis bands and free-probes are indicated with arrows. His protein was used as a negative control. **C)** Gene expression analysis of *PME6* in *myb156* mutants. The data are presented as means ± Se (n = 3 plants per genotype). **Significant at *P* < 0.01 compared with WT (#, representing the control for comparison) based on Student's *t*-test. Black dots represent the individual data for each plant replicate. **D)** Transcriptional repression activity of MYB156 on the *PME6* promoter with a dual LUC assay. Relative LUC activities were measured after co-transformation with the reporter vector (*ProPME6:LUC*) and the effector vector (*Pro35S:MYB156*). The empty vector (pGreen-SK) was used as control. The data are presented as means ± Se (n = 8 replicate reactions, each based on an individual transfected plants). Black dots represent the individual data for each reaction. ** Significant at *P* < 0.01 compared with control (#, representing the control for the comparison) using Student's *t*-test. **E)** Expression levels of *MYB156* and *PME6* in *MYB156* and/or *PME6*-overexpressing transgenic plants by reverse transcription quantitative PCR (RT-qPCR). DOE03 and DOE38 are the transgenic lines in which both *MYB156* and *PME6* are overexpressed. The data are presented as means ± Se of 3 plants per genotype. Black dots represent the individual data for each plant replicate. **F)** Water loss rate in *MYB156* and/or *PME6*-overexpressing transgenic plants. The data are presented as means ± Se of 10 plants per genotype. **G)** Stomatal conductance response to changing CO_2_ concentration in *MYB156* and/or *PME6*-overexpressing transgenic plants. The data are presented as means ± Se of 10 plants per genotype. Note that the defective stomatal dynamic range in the *MYB156* overexpression (OE) 15 (*MYB156*OE15) was recovered by *PME6* overexpression in DOE03.

To learn whether *PME6* is a direct target of MYB156, yeast 1-hybrid (Y1H) and electrophoretic mobility shift assay (EMSA) were performed. The Y1H assays showed that MYB156 could bind to an 83-bp fragment in the *PME6* promoter (P1 fragment) that contains an AC-III element (Fig. 5A). The EMSA confirmed that MYB156 could specially bind to the AC-III element of the *PME6* promoter (Fig. 5B). The RT-qPCR analysis showed that *PME6* expression was decreased in *MYB156*OEs and increased in *myb156* mutants compared with WT (Figs. 4F and 5C). Accordingly, the dual-LUC assay validated that the expression of *PME6* was repressed by MYB156 (Fig. 5D). These results suggest that *PME6* is a direct target of MYB156 and MYB156 acts as a transcriptional repressor to negatively regulate the expression of *PME6*.

To further validate the epistatic relationship between *MYB156* and *PME6*, we overexpressed *PME6* in *MYB156*OE15 as well as in the WT background and obtained 2 transgenic lines that constitutively expressed both *MYB156* and *PME6*, named DOE03 and DOE38, and 2 *PME6*-overexpressing lines, named *PME6*OE12 and *PME6*OE13. The RT-qPCR analysis showed that *PME6* was highly expressed in those transgenic lines (Fig. 5E). We observed the defects of the stomata reflected by the water loss from the detached leaves, and changes of *g_s_* dynamics to shifts of CO_2_ conditions in *MYB156*OE15 (Fig. 5, F and G, the dark-green line) were recovered by the overexpression of *PME6* (Fig. 5, F and G, the light-green line), suggesting that *PME6* is genetically epistatic to *MYB156*.

**Figure 6.**
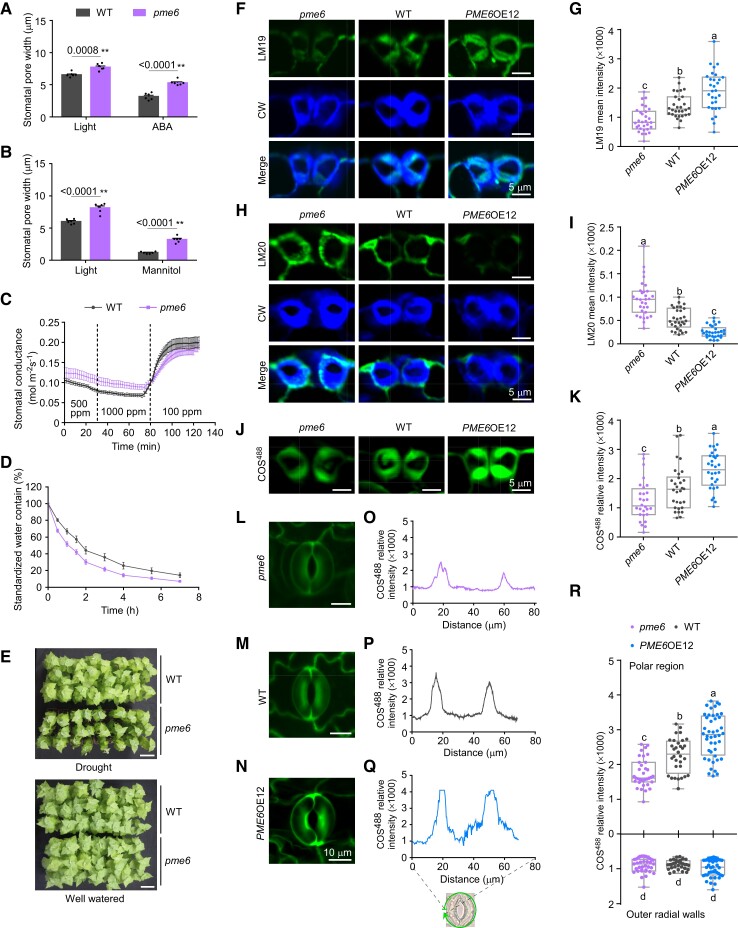
Knocking-out of *PME6* Impairs Stomatal Function and Alters the Degree of Methylesterification of Pectic Homogalacturonan (HG) in Guard Cells. **A)** Stomatal response to light and abscisic acid (ABA) treatment in wild-type (WT) and *pme6*. Stomatal opening was induced under light for 2 h, following which stomatal closure was induced by 20 mm ABA for 4 h. The data are presented as means ± Se for six plants per genotype per treatment. Each plant was measured for the mean pore width of 20 stomata, which were randomly sampled from two fully expanded LPI6 and 7 leaves (LPI, Leaf Plastochron Index, which was used as an indicator of the leaf age, with LPI6 and 7 respectively representing the sixth and seventh leaf from the plant top). Black dots represent the individual data for each plant replicate. Asterisks indicate significant differences (**P < 0.01) compared to WT plants, as determined by Student's t-test. The precise *P* values are also provided. **B)** As in **(A)** but treated with 1 M mannitol for 4 h to induce stomatal closure. **C)** Stomatal conductance response to changing CO_2_ concentration. The data are presented as means ± Se of 10 plants per genotype. Note the slightly decreased stomatal dynamic range in response to the changing CO_2_ concentration in *pme6* compared with WT. **D)** The detached leaves of the *pme6* mutant showed faster water evaporation. Time-course of cumulative water loss from the leaves via transpiration was examined at the indicated time points after the leaves were detached. The data are presented as means ± Se of 10 plants per genotype. **E)***pme6 Populus* showed a sensitive phenotype to drought stress. For each genotype, 21 plants were grown. The phenotype of WT and *pme6* was recorded after withholding water for 9 days, using well-watered plants as the control. The experiments were conducted three times, with similar results obtained each time. Bar = 10 cm. **F)** Representative de-esterified HG images of guard cell cross-sections of *pme6*, WT, and *PME6* overexpression (OE) 12 (*PME6*OE12) plants. The cross-sections of guard cells from LPI6 leaves of 2-month-old plants were immunolabeled with LM19 (in green) and counterstained with Calcofluor White (CW) (in blue) to show guard cell walls. Bar = 5 *μ*m. **G)** Quantification of LM19 labeling intensity in cross-sections of *pme6*, WT, and *PME6*OE12 guard cells. **H, I)** Same as in **F, G)**, respectively, but labeled with LM20, which indicates highly methylesterified HG. Bar = 5 *μ*m. **J-K)** Same as in **(F, G)**, respectively, but labeled with COS^488^, which indicates demethylesterified HG. Bar = 5 *μ*m. **L-Q)** The distribution of de-esterified HG (labeled with COS^488^) around the stomatal circumference in *pme6*, WT, and *PME6*OE12. Representative images of COS^488^ labeling in *pme6*, WT, and *PME6*OE12, are shown in **(L)**, **(M)** and **(N)**. The distribution of COS^488^ signals around the circumference of the stomatal complex of *pme6*, WT, and *PME6*OE12 is shown in **(O), (P),** and **(Q)**, with the start point set to the equator (as shown in the schematic). Note the decreased COS^488^ signals at the stomatal poles in *pme6*, but increased signals in *PME6*OE12 compared with WT. Bar = 5 *μ*m. **R)** Quantification of the COS^488^ signals in the polar regions and the outer radial walls around the stomatal circumference of *pme6*, WT, and *PME6*OE12. For the box-and-whisker plots in **(G)**, **(I)**, **(K)**, and **(R)**, whiskers extend to min and max, box boundaries represent the 25th percentile (upper) and 75th percentile (lower), the lines inside boxes represent medians, and dots represent the individual data for each pair of guard cells. At least 30 pairs of guard cells from three different plants per genotype were investigated. Different letters indicate statistically significant differences across genotypes, while the same letter indicates no significant difference according to one-way ANOVA Duncan's (D) test (*P* < 0.05).

To elucidate the role of PME6 in the guard cells, 2 types of transgenic plants were generated. The first type expressed a GUS reporter driven by the *PME6* promoter (*ProPME6*), while the 2nd type expressed Cas9/sgRNA with a target sequence specific to *PME6* (Supplemental Fig. S2, A and B). The GUS histochemical staining showed that *PME6* was highly expressed in the guard cells. Stomatal functions were then assayed in the CRISPR/Cas9-mediated *pme6* mutant. A larger degree of opening after light induction and less closing degree after ABA, mannitol, and higher CO_2_ concentration (1,000 ppm) treatments were observed in the *pme6* mutant compared with WT (Fig. 6, A to C). Accordingly, water loss from the detached leaves was more rapid in *pme6* than in WT (Fig. 6D) and *pme6* plants showed a sensitive phenotype to drought stress (Fig. 6E). The similar phenotype between the *pme6* mutant and *MYB156*OEs (Figs. 3 and 6, A to E) further supports the idea that MYB156 negatively regulates *PME6* in guard cell functioning.

To investigate how the degree of HG methylesterification of guard cell walls plays roles in stomatal functioning, we probed the guard cells of WT and *PME6* transgenic plants with several antibodies and probes that recognize different forms of HG. LM19 and COS^488^ interact with low methylesterified HG (Verhertbruggen et al. 2009; Mravec et al. 2014), while LM20 recognizes high methylesterified HG (Verhertbruggen et al. 2009). Negative controls for immunolabeling the guard cells of WT and *PME6* transgenic plants did not show specific signals (Supplemental Fig. S2C, D). By applying these antibodies/probes to leaf cross-sections, we found that COS^488^ and LM19 labeling intensities were significantly higher and LM20 labeling intensities were significantly lower, in *PME6*OE12 guard cells than in WT guard cells (Fig. 6, F to K). This suggests that *PME6*OE guard cell walls contain more demethylesterified HG (available for COS^488^ and LM19 binding), but less methylesterified HG, than WT controls. Conversely, in *pme6* guard cells, LM19 and COS^488^labeling intensities were significantly lower and LM20 labeling intensities were significantly higher, than in WT control (Fig. 6, F to K). This indicates that *pme6* guard cell walls contain less demethylesterified HG, but more methylesterified HG, than WT controls.

Interestingly, when we performed COS^488^ labeling on intact guard cells of WT and *PME6*-manipulated transgenic plants, we found COS^488^ signals enriched in the polar regions of the stomatal complex in WT *Populus* (Fig. 6, M, P, and R). These signals decreased significantly in the *pme6* mutants (Fig. 6, L, O, and R) but increased significantly in *PME6*OE12 (Fig. 6, N, Q, and R). This decrease in *pme6* mutants appears to occur specifically in the polar regions around the circumference of the stomatal complex, but not at the outer radial wall (Fig. 6R). This suggests that the loss of *PME6* decreased the abundance of demethylesterified HG in the polar regions of the *pme6* mutants and then altered the pattern of HG epitopes around the circumference of the stomatal complex.

The highly anisotropic nature of the guard cell wall in composition and molecular architecture, as well as the spatial distribution of wall properties (including thickness and biomechanics) (Marom et al. 2017; Yi et al. 2019), complicates the interpretation of data on HG epitope labeling. To understand the roles of HG epitopes in stomatal functioning, their spatial dynamics need to be monitored during stomatal opening and closing. As a result of these technical challenges, our detailed understanding of how the molecular architecture of guard cell wall plays a role in guard cell function is still somewhat limited (Altartouri and Geitmann 2015; Woolfenden et al. 2018). However, when taken together, the data presented in Fig. 6, L to R, support the insight into the relationship of mechanics and function in stoma, which highlights the crucial role of demethylesterified HG-based stomatal polar stiffening in stomatal functioning (Carter et al. 2017). These suggests that although the degree of HG methylesterification was modulated throughout the guard cell walls in *PME6*-manipulated transgenic plants (Fig. 6, F to K), PME6 seems to function in stomatal function mainly through regulating HG methylesterification in the polar regions of the stomata.

### MYB156 regulates HG methylesterification and wall stiffness in the polar regions of the guard cell walls for stomatal functioning

**Figure 7.**
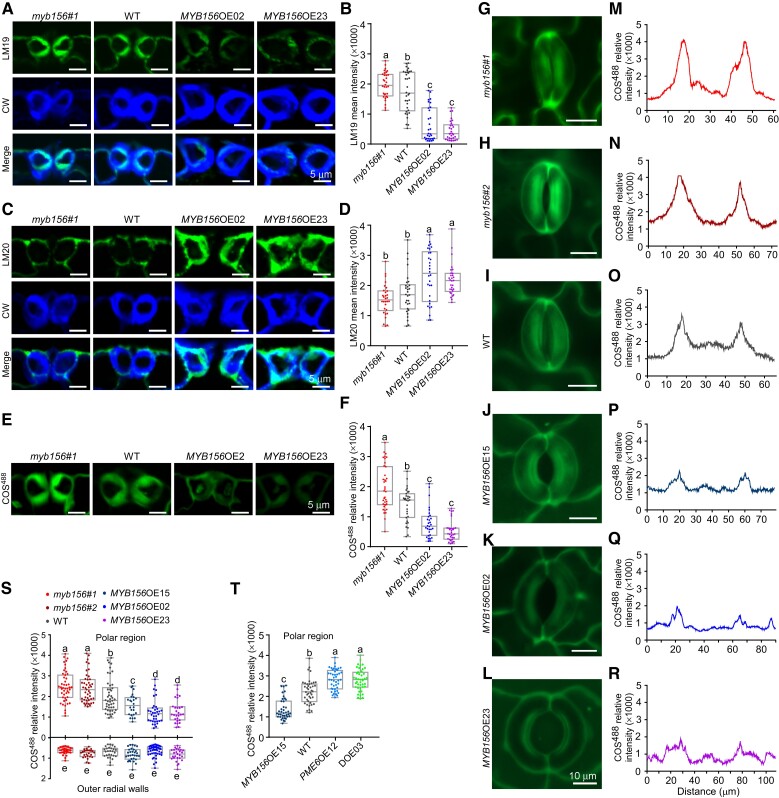
Changes in *MYB156* and/or *PME6* Expression Alter HG Methylesterification Status in Guard Cells. **A)** Representative de-esterified HG images of cross-sections of *myb156 #1*, wild-type (WT), and *MYB156* overexpression (OE) 02 and 23 guard cells. The cross-sections of guard cells from LPI6 leaves (LPI, Leaf Plastochron Index, which was used as an indicator of the leaf age, with LPI6 representing the sixth leaf from the plant top) of 2-month-old plants were immunolabeled with LM19 (in green) and counterstained with CW (in blue) to show guard cell walls. Bar = 5 *μ*m. **B)** Quantification of LM19 labeling intensity in cross-sections of *myb156 #1*, WT, *MYB156*OE02 and *MYB156*OE23 guard cells. n = 30 pairs of guard cells from three different plants per genotype. **C, D)** Same as in **(A, B)**, respectively, but labeled with LM20, which indicates highly methylesterified HG. Bar = 5 *μ*m. **E)** Representative images of COS^488^ labeling of cross-sections of *myb156 #1*, WT, *MYB156*OE02 and *MYB156*OE23 guard cells. Bar = 5 *μ*m. **F)** Same as in (**B**), but labeled with COS^488^. **G-L)** COS^488^-labeled stomatal images. Note that the abundance of de-esterified HG at the stomatal poles is increased in *myb156#1***(G)** and *myb156 #2***(H)**, but greatly diminished in *MYB156*OE15 **(J)**, *MYB156*OE02 **(K)**, and *MYB156*OE23 **(L)** compared with WT **(I)**. Bars = 10 *μ*m. **M-R)** Distribution of COS^488^ signals around the circumference (as shown in schematic in Figure 6) of the corresponding stomatal complex shown in **(G-L)**. **S)** Quantification of the COS^488^ signals at the two peaks of stomatal poles and the outer radial walls in *myb156* mutants, WT, and *MYB156*OEs. **T)** Less de-esterified HG in the polar regions of the stomata of *MYB156*OE15 was recovered by *PME6* overexpression in the transgenic line DOE03. DOE03 is the transgenic line in which both *MYB156* and *PME6* are overexpressed. At least 30 pairs of guard cells were investigated in **(S)** and 25 pairs of guard cells in **(T)**, from three different plants per genotype. For the box-and-whisker plots in **(B)**, **(D)**, **(F)**, **(S)**, and **(T)**, whiskers extend to min and max, box boundaries represent the 25th percentile (upper) and 75th percentile (lower), the lines inside boxes represent medians, and dots represent the individual data for each pair of guard cells; Different letters indicate statistically significant differences across genotypes, while the same letter indicates no significant difference according to one-way ANOVA Duncan's (D) test (*P* < 0.05).

The above results demonstrated that MYB156 functions in stomatal dynamics through downregulating the expression of *PME6*. To determine whether MYB156 controls the degree of HG methylesterification of guard cell walls, labeling with COS^488^, LM19, and LM20 was performed on *MYB156*-manipulated transgenic plants. Negative controls for immunolabeling the guard cells of *MYB156*-manipulated transgenic plants did not show specific signals (Supplemental Fig. S3). By applying these antibodies/probes to cross-sections of *MYB156*OE02 and *MYB156*OE23 guard cells, we found that COS^488^ and LM19 labeling intensities were significantly lower and LM20 labeling intensities were significantly higher than those in WT guard cells (Fig. 7, A to F). This suggests that *MYB156*OE guard cell walls contain less demethylesterified HG, but more methylesterified HG, than WT controls. In *myb156* guard cells, COS^488^ and LM19 labeling intensities were significantly higher than those in WT controls but LM20 labeling intensities did not differ from the control (Fig. 7, A to F). This suggests that *myb156* guard cell walls contain more demethylesterified HG. Overall, the data in Fig. 7, A to F, indicate that MYB156 controls the degree of HG methylesterification of guard cell walls.

Given the significance of demethylesterified HG-based polar stiffening on guard cell function, we performed COS^488^ probing on the intact guard cells of *MYB156*-manipulated transgenic plants to detect the HG epitope pattern around the stomatal complex circumference (Fig. 7, G to T). We found that the COS^488^ signals, particularly in the polar region, were higher in the *myb156* mutants and lower in *MYB156*OEs than in WT. The signal intensity of polar regions (which reflects the local abundance of de-esterified HG) was negatively associated with the expression level of *MYB156* among the detected genotypes (Figs. 3A and 7S). Moreover, the abundance of de-esterified HG in the polar region appears to control stomatal dynamic ranges. The smallest dynamic ranges were observed with the lowest COS^488^ intensities (reflecting less de-esterified HG) of the polar region in *MYB156*OE02 and *MYB156*OE23 guard cells, while the largest dynamic ranges were observed with the highest COS^488^ intensities (reflecting more de-esterified HG) of the polar region in *myb156 #1* and *myb156 #2* guard cells (Figs. 2C, 3D, and 7S). These findings emphasize the critical role of demethylesterified HG-based polar stiffening in guard cell functioning.

Furthermore, less de-esterified HG (illustrated by lower COS^488^ intensities) in the polar regions of the stomata of *MYB156*OE15 was recovered by *PME6* overexpression in the transgenic line DOE03 (Fig. 7T), as was the limited stomatal dynamics (Fig. 5G). These data suggest that MYB156 fulfills its function in stomatal dynamics through regulating the enrichment of de-esterified HG in the polar regions of the guard cell walls.

**Figure 8.**
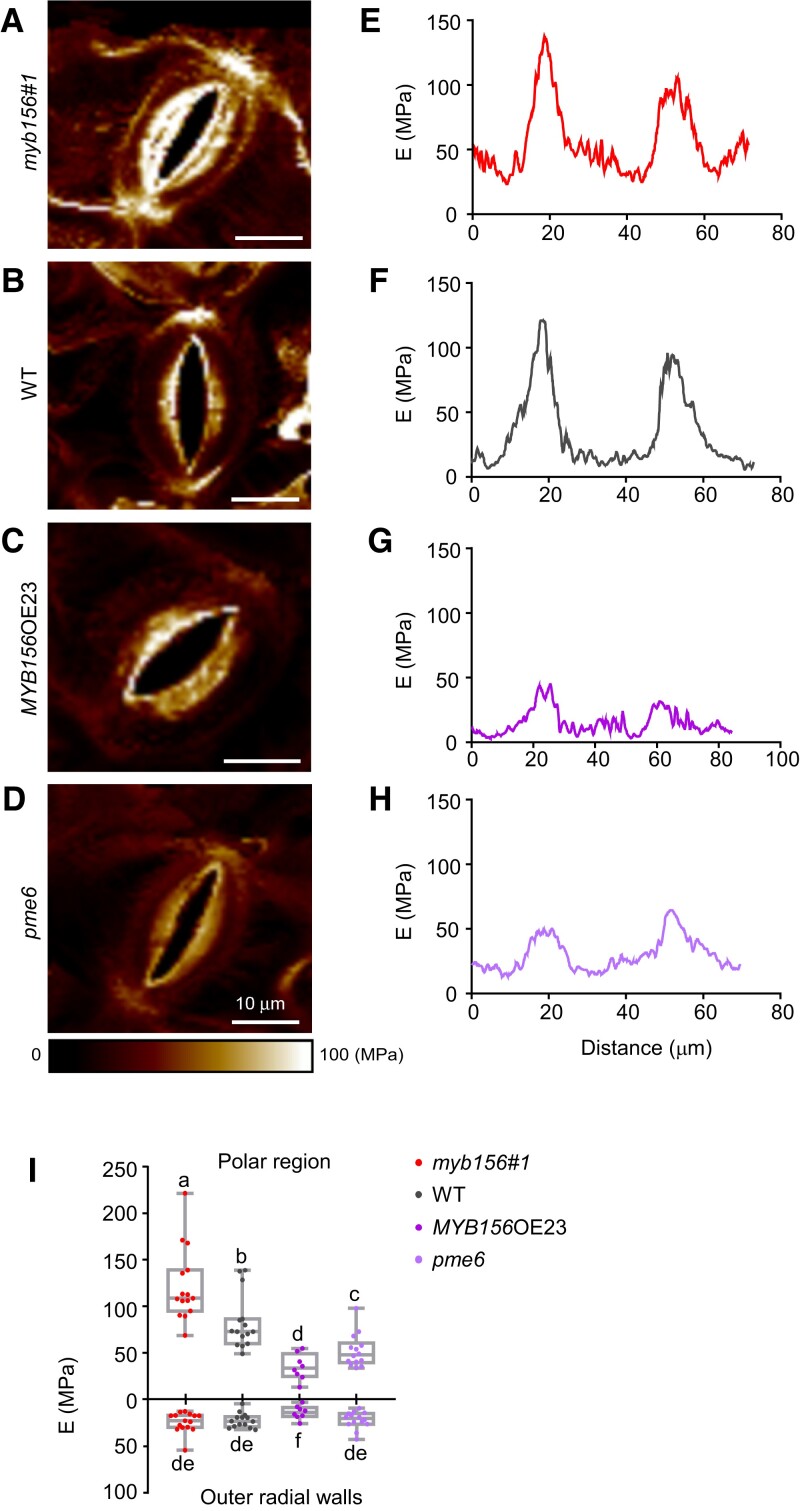
Changes in *MYB156* or *PME6* Expression Alter Stiffness in the Polar Regions of the Guard Cells. **A-D)** Force map of the guard cell walls of *myb156 #1***(A)**, wild-type (WT) **(B)**, *MYB156* overexpression (OE) 23 **(C)**, and *pme6***(D)**, as measured by atomic force microscopy. Bar = 10 *μ*m. **E-H)** Distribution of the apparent modulus (E) around the stomatal circumference (as shown in schematic in Figure 6) of the corresponding stomatal complex shown in **(A-D)**. Two peaks of E with various extents were observed at the poles of the stomatal complex. **I)** Quantification of the E value at the two peaks of stomatal poles and the outer radial wall in *myb156 #1*, WT, *MYB156*OE23 and *pme6*. At least 10 pairs of guard cells from three different plants per genotype were investigated. Whiskers extend to min and max, box boundaries represent the 25th percentile (upper) and 75th percentile (lower), the lines inside boxes represent medians, and dots represent the individual data for each pair of guard cells. Different letters indicate statistically significant differences across genotypes, while the same letter indicates no significant difference according to one-way ANOVA Duncan's (D) test (*P* < 0.05).

To investigate the effect of the abundance of de-esterified HG in the polar regions on the stomatal mechanics in *MYB156* or *PME6*-manipulated transgenic *Populus*, we performed atomic force microscopy (AFM) in the genotypes generated in this study. Force maps were generated by probing the abaxial surface of the leaves after mannitol treatment (Fig. 8, A to H), where cell wall stiffness was indicated by apparent modulus values (*E*). In WT plants, the *E* value peaked at the stomatal poles, as shown in the force map (Fig. 8B) and the quantitative analysis of *E* around the circumference of the stomatal complex (Fig. 8F). In *myb156 #1*, the peak signals became higher than those in WT (Fig. 8, A, B, E, F, and I), whereas in *MYB156*OE23, they were significantly reduced compared with those in WT (Fig. 8, B, C, F, G, and I). Similar to that in *MYB156*OE23, the peak signals in the *pme6* mutant were also reduced (Fig. 8, B, D, F, H, and I), reflecting the opposite effects exerted by MYB156 and PME6 on wall stiffness. In line with the pattern of demethylesterified HG around the stomatal circumference, wall stiffness was also modulated particularly in the polar regions of the stomata (Fig. 8I), suggesting that the enrichment of de-esterified HG dictates wall stiffness in the guard cells. Overall, these data indicate that the increase of de-esterified HG in the polar regions would explain the localized stiffer wall in *myb156*, which was associated with enhanced stomatal dynamics, and in *MYB156*OEs, the defective stomatal dynamics were accompanied by weakened polar stiffness resulting from the decrease of de-esterified HG in those regions.

### Polar stiffening is important for the normal morphology and proper dynamics of stomata

**Figure 9.**
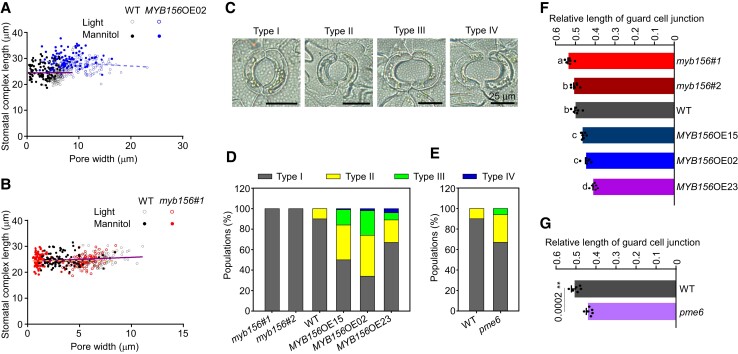
Polar Stiffening is Required for Maintaining the Normal Guard Cell Morphology during Stomatal Opening. **A, B)** Stomatal complex length remains constant during stomatal movement. Stomatal opening was induced under light for 2 h, following which stomatal closing was induced by mannitol treatment of 4 h (n = 100 stomata from six different plants per genotype). Regression analysis showed no linear relationship between complex length and pore width in wild-type (WT, purple full line in **A**, **B**), *MYB156* overexpression (OE) 02 (*MYB156*OE02, blue dashed line in **A**), and *myb156 #1* (red dashed line in **B**), each with a low Pearson's r values of 0.01108 (for WT in **A**), −0.1234 (for *MYB156*OE02 in **A**), 0.1526 (for WT in **B**), and 0.0245 (for *myb156 #1* in **B**). **C**) Four types of stomatal morphologies according to the ratio of pore width to its length (R^W/L^) in stomatal open states. Type I, R^W/L^ < 0.8; type II, 0.8 ≤ R^W/L^ < 1.0; type III, R^W/L^ ≥ 1.0; type IV, with separated sister guard cells. Representative images of different types of stomata are shown. Bar = 25 *μ*m. **D**, **E)** The proportion of different types of stomata in the indicated genotypes. About 100 stomata from six different plants per genotype were examined, with each plant contributing approximately 15-20 stomata, which were randomly sampled from two fully expanded LPI6 and 7 leaves (LPI, Leaf Plastochron Index, which was used as an indicator of the leaf age, with LPI6 and 7 respectively representing the sixth and seventh leaf from the plant top). **F**, **G**) The relative length of the guard cell junction at stomatal open states in the indicated genotypes. The relative junction length was recorded as a ratio of junction length to the stomatal complex length. The data are presented as means ± Se of six plants per genotype. Each plant was measured for the mean relative length of the guard cell junction of approximately 15-20 stomata, which were randomly sampled from two fully expanded leaves (LPI6 and 7). Black dots represent the individual data for each plant replicate. Different letters indicate statistically significant differences across genotypes, while the same letter indicates no significant difference according to one-way ANOVA Duncan's (D) test (*P* < 0.05) in **(F)**. **Significant at *P* < 0.01 (the precise *P* values provided) compared with WT based on Student's *t*-test in **(G)**.

Given the essential role of the cell wall in controlling the shape change of guard cells during stomatal movement, we next investigated the effect of polar stiffening on stomatal morphology in the genotypes generated in this study. We first measured the stomatal complex length of WT, *myb156 #1*, and *MYB156*OE02 during stomatal movement and found that the stomatal complex lengths were kept constant from the closed state to the open state (Fig. 9, A and B). Other authors also reported the similar results and claimed that the polar stiffness restricts the increase of stomatal complex length during opening (Rui and Anderson 2016; Carter et al. 2017). Our result suggests that the limited polar stiffening in *MYB156*OE02 still can prevent the increase in the stomatal complex length during stomatal opening.

To further explore the effect of polar stiffening on stomatal morphology, we investigated stomatal geometries in the open state for all genotypes generated in this study. The ratio of pore width to pore length (*R*^W/L^) and relative junction length (recorded as the ratio of junction length to stomatal complex length) were used to characterize the stomatal morphology. According to the value of *R*^W/L^, stomata were classified into 4 types: type I (*R*^W/L^ < 0.8), type II (0.8 ≤ *R*^W/L^ < 1.0), type III (*R*^W/L^ ≥ 1.0), and type IV (with separated sister guard cells) (Fig. 9C). WT plants contained only type I and type II stomata, suggesting the pore width was no larger than its length at the open state (Fig. 9D). By contrast, in some of the *MYB156*OEs and *pme6* stomata, the pore width displayed an excess increase during stomatal opening, reflected by the groups of type III stomata (where the pore width is larger than its length) and type IV stomata characterized by separated sister guard cells (Fig. 9, D and E), suggesting that cell adhesion was damaged. Consistently, the relative length of the guard cell junction was significantly shorter in *MYB156*OEs and *pme6* stomata and was longer (or the same) in *myb156* stomata, compared with that of WT stomata (Fig. 9, F and G). These data imply that polar stiffening is required for maintaining the adhesion of sister guard cells and normal guard cell morphology during stomatal opening.

**Figure 10.**
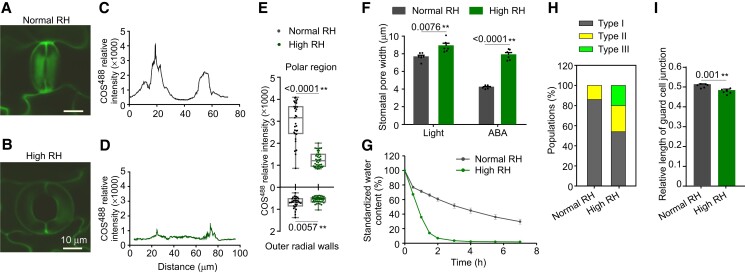
High Relative Air Humidity Induces the Stomatal Malfunction through Decreasing HG De-methylesterification in the Polar Regions. **A**-**G)** High relative air humidity (RH) induced a decrease of HG de-methylesterification in the stomatal polar regions and stomatal malfunction. Representative COS^488^-labeled stomatal images of wild-type plants grown under normal and high RH shown in **(A)** and **(B)**, respectively, and distribution of COS^488^ signals around the circumference of the stomatal complex shown in **(C, D)**. Quantification of the COS^488^ signals in the polar regions and in the outer radial walls under normal and high RH shown in **(E).** Whiskers extend to min and max, box boundaries represent the 25th percentile (upper) and 75th percentile (lower), and the lines inside boxes represent medians. At least 30 pairs of guard cells from three different plants per treatment were investigated. The limited stomatal closure under high RH to abscisic acid (ABA) is shown in **(F).** The data are presented as means ± Se of six plants per treatment. Each plant was measured for the mean pore width of approximately 15-20 stomata, which were randomly sampled from two fully expanded LPI6 and 7 leaves (LPI, Leaf Plastochron Index, which was used as an indicator of the leaf age, with LPI6 and 7 respectively representing the sixth and seventh leaf from the plant top). Black dots represent the individual data for each plant replicate. Faster water evaporation from the detached leaves of plants grown under high RH than that under normal RH is shown in **(G).** The data are presented as means ± Se of at least 10 plants per treatment. Bar = 10 *μ*m. **H)** The proportion of different types of stomata in wild-type plants grown under normal and high RH. About 100 stomata from six different plants per treatment were examined, with each plant contributing approximately 15-20 stomata, which were randomly sampled from two fully expanded leaves (LPI6 and 7). **I)** The relative length of the guard cell junction at the stomatal open state in wild-type plants grown under normal and high RH. The relative junction length was recorded as a ratio of junction length to the stomatal complex length. The data are presented as means ± Se of six plants per treatment. Each plant was measured for the mean relative length of the guard cell junction of approximately 15-20 stomata, which were randomly sampled from two fully expanded leaves (LPI6 and 7). Black dots represent the individual data for each plant replicate. Asterisks in **(E)**, **(F)**, and **(I)** indicate significant differences (*P < 0.05; **P < 0.01) compared with control guard cells from plants under normal RH conditions, as determined by Student's t test. The precise *P* values are also provided.

To explore the potential physiological role of polar stiffening in plants, we investigated whether the stomatal malfunction induced by high relative air humidity (RH ≥ 85%) (Fanourakis et al. 2020) was related to the alteration of the polar stiffening of the guard cells. For this, we used *Populus* plants cultivated on Murashige & Skoog (MS) medium in sterile jars, wherein the RH was nearly saturated. Since the enrichment of de-esterified HG dictates wall stiffness in guard cells (Figs. 7M and 8I), we analyzed the abundance of de-esterified HG in those plants using a COS^488^ probe. Under high RH, the COS^488^ signals decreased significantly in the stomatal polar regions compared with those under normal RH (Fig. 10, A to E), suggesting a decrease of HG de-methylesterification. After light induction, those stomata displayed a larger aperture than those under normal RH and nearly did not close in response to the ABA closing trigger (Fig. 10F). Consequently, the water loss rates were higher in those plants (Fig. 10G). As in *MYB156*OEs, a proportion of type III stomata was observed in plants grown under high RH, but not in those grown under normal humidity (Fig. 10H). Also, the relative length of the guard cell junction was reduced compared with those of the plants grown under normal humidity (Fig. 10I). These results suggest that high RH-induced stomatal malfunction is mediated by decreasing the abundance of de-esterified HG in the polar regions, thus impairing the normal guard cell morphology during stomatal movement. These data further imply that the polar stiffening of stomata is modulated for the environmental adaptation of plants.

### Normal *MYB156* expression is required for leaf growth in *Populus*

**Figure 11.**
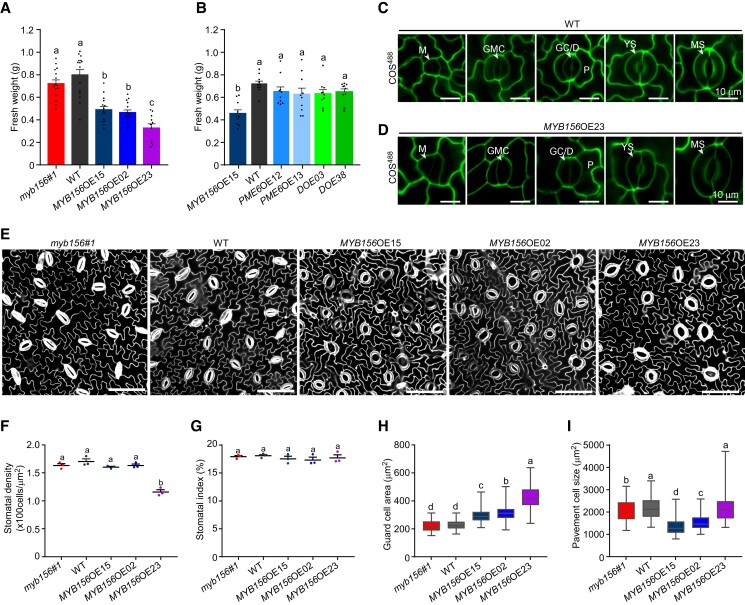
*MYB156* Overexpression Affects Plant Growth. **(A)** Quantification of fresh weight of *myb156 #1*, wild–type (WT), *MYB156* overexpression (OE) 15, 02, and 23 leaves. The fresh weight of LPI6 and LPI7 leaves (LPI, Leaf Plastochron Index, which was used as an indicator of the leaf age, with LPI6 and 7 respectively representing the sixth and seventh leaf from the plant top) from 2–month–old plants were measured. The data are presented as means ± Se of 15 plants per genotype. Black dots represent the individual data for each plant replicate. **(B)** Quantification of fresh weight of *MYB156*OE15, WT, *PME6*OE12, *PME6*OE13, DOE03, and DOE38. The fresh weight of LPI6 and LPI7 leaves from 2–month–old plants were measured. The data are presented as means ± Se of 10 plants per genotype. Black dots represent the individual data for each plant replicate. **(C, D)** Representative image of COS^488^–labeled stomata of various developmental stages of WT **(C)** and *MYB156*OE23 plants **(D)**. The younger leaves of WT and *MYB156*OE23 (less than 2 cm in length) were used for labeling. M: meristemoid (triangular); GMC: guard mother cell (oval); GC/D, guard cell undergoing development; YS: young stomata (with a length approximately equal to its width); MS: mature stomata (with a larger length compared to its width); P: pavement cell. Bar = 10 *μ*m. **(E)** Representative image of PI–stained epidermal cells of LPI6 leaves of 2–month–old *myb156 #1*, WT, *MYB156*OE15, *MYB156*OE02, and *MYB156*OE23. Bar = 50 *μ*m. **(F–I)** Quantification of stomatal density **(F)**, stomatal index **(G)**, guard cell area **(H)**, and pavement cell size **(I)** in 2–month–old *myb156 #1*, WT, *MYB156*OE15, *MYB156*OE02, and *MYB156*OE23. For **(F)** and **(G)**, n = three individual plants with five fields of each for quantification of stomatal density and index. Stomatal index = number of stomata (ns) divided by the sum of stomata number and pavement cell number (np) per field (ns/(ns + np)). The data are presented as means ± Se of 3 plants per genotype, with each dot representing the individual data for each plant replicate. n = 100 guard cells from six individual *Populus* plants per genotype in **(H)**, and n = 100 pavement cells from three individual *Populus* plants per genotype in **(I)**. Whiskers extend to min and max, box boundaries represent the 25th percentile (upper) and 75th percentile (lower), the lines inside boxes represent medians; Different letters indicate statistically significant differences across genotypes, while the same letter indicates no significant difference according to one–way ANOVA Duncan's (D) test (*P* < 0.05).

Given the strong expression of *MYB156* during stomatal differentiation and its age-dependent expression pattern (Fig. 1), we investigated the function of MYB156 in plant growth and development by examining the fresh weight of mature leaves of different genotypes. While *myb156* leaves showed a comparable average fresh weight with WT leaves, the average fresh weight of *MYB156*OE leaves was much lower than that of WT leaves. The extent of decrease in leaf fresh weight increased with increasing expression of *MYB156* among the 3 *MYB156*OE lines, with the highest *MYB156* expression level being paired with the lowest fresh weight in *MYB156*OE23 plants (Figs. 3A and 11A). Similar results were observed for leaf size among the 3 *MYB156*OE lines (Supplemental Fig. S4). Additionally, we observed that the limited leaf growth of *MYB156*OE15 was rescued by *PME6* overexpression in the transgenic lines DOE03 and DOE38 (Fig. 11B). These results suggest that MYB156 functions in plant growth and development by modulating the degree of demethylesterification of cell wall HG through downregulating the expression of *PME6*.

To explain how MYB156 affects leaf development and growth at the cellular level, we investigated the HG methylesterification status of guard cell walls during stoma differentiation. We also measured the size of guard cells and pavement cells, as well as stomatal density (number of stomata per unit area) and stomatal index (number of stomata divided by the sum of stomata and pavement cells) in mature leaves of different genotypes. During stomatal differentiation, the demethylesterified HG-involved cell wall polarization occurred before the symmetric division of guard mother cells and manifested as polar stiffening of stomata later on in WT plants (Fig. 11C). This polarized pattern of the cell wall was weakened in *MYB156*OE23 guard mother cells and stomata of various developmental stages but was still visible (Fig. 11D). In mature leaves, stomatal patterning, stomatal density, and stomatal index were mostly unaffected in *MYB156*-manipulated transgenic lines, except for *MYB156*OE23 plants, which showed a significant decrease in stomatal density (Fig. 11, E to G). Compared to WT controls, all 3 *MYB156*OE lines had larger guard cells, while their pavement cells did not show an enlarged phenotype (Fig. 11, H and I). *MYB156*OE15 and OE02 plants even possessed smaller pavement cells, suggesting that MYB156 affects cell expansion in a cell type–specific manner. In the case of *MYB156*OE23, the considerable increase in guard cell size combined with unchanged pavement cell size implied that the dramatic reduction in *MYB156*OE23 leaf size can be attributed to the reduction of cell proliferation. This suggests that MYB156 affects cell proliferation as well as cell expansion.

## Discussion

Herein, we report that the *Populus* transcription factor MYB156 participates in the regulation of HG-based polar stiffening in the guard cells through the downregulation of *PME6* (Figs. 5, 7, and 8). We found that the demethylesterified HG-based stiffness in the polar regions was enhanced in *myb156* mutants and weakened in *MYB156*OEs (Fig. 8), indicating that MYB156 negatively regulates polar stiffening in the guard cells. As a result, the stomata of *myb156* opened and closed more quickly over a larger dynamic range than the WT stomata in response to changing CO_2_ concentration (Fig. 2C), whereas the stomata of *MYB156*OEs responded to the stimuli more slowly with a smaller dynamic range than the WT stomata (Fig. 3D). Furthermore, the dynamic ranges were negatively associated with the gene expression levels among the 3 *MYB156*OE lines (Fig. 3, A and D), illustrated by the smallest dynamic range being paired with the highest *MYB156* expression level in *MYB156*OE23. These results suggest that polar stiffening is essential for stomatal dynamics.

### Polar stiffening is involved in stomatal movements

Stomatal polar stiffening has been reported in other plant species, such as Arabidopsis (*Arabidopsis thaliana*), tomato (*Solanum lycopersicum*), and maize (*Zea mays*) (Carter et al. 2017). It is proposed to be involved in stomatal opening through preventing the increase in guard cell complex length. In this study, we found that weakened polar stiffening was still able to prevent the increase of guard cell complex length during stomatal opening in a *MYB156*OE line (Fig. 9, A and B) but it reduced the ability of stomata to maintain normal stomatal morphology and the adhesion of the sister guard cells during stomatal opening (Fig. 9, C to G).

We propose that this reduction in polar stiffening was caused by a decrease in the abundance of demethylesterified HG in the polar region of *MYB156*OE plants. This finding is consistent with the role of demethylesterified HG in cell adhesion, as characterized in the *quasimodo1* mutant. This mutant is defective in galacturonosyltransferase activity and exhibits reduced cell adhesion. The fact that this mutant had a greater decrease in de-esterified HG content (over a 50% reduction) than in pectin abundance (about a 25% reduction in galacturonic acid levels), compared with WT plants, suggests that de-esterified HG, rather than HG abundance, is what holds cells together like cement (Bouton et al. 2002; Mravec et al. 2014). These results suggest a key role of polar stiffening in stomatal opening through holding the sister guard cells together, thereby preventing excess increases in pore width during stomatal opening. The importance of the junction region between 2 sister guard cells during stomatal opening has also been proposed by other authors (Aylor et al. 1973; Yi et al. 2019). In addition, we found that the polar stiffening of the stoma was also involved in stomatal closing (Fig. 3) and was physiologically modulated (Fig. 10).

### Spatial regulation of PME controls stomatal polar stiffening

During pollen germination, PMEs act specifically on the cell walls near the apex of the pollen tube through PME polar trafficking (Chebli et al. 2012; Wallace and Williams 2017). During hypocotyl elongation (Peaucelle et al. 2015) and organ initiation (Peaucelle et al. 2011; Braybrook and Peaucelle 2013; Wachsman et al. 2020), the activation of PME activity is also assumed to take place at specific microdomains of the apoplast. Several purified PMEs from various sources have been shown to be more active at neutral to alkaline pH and under optional cations (Do Amaral et al. 2005; Verlent et al. 2007; Hewezi et al. 2008; Pelletier et al. 2010; Dixit et al. 2013; Senechal et al. 2015). Meanwhile, the specific pH microdomain that is favorable for PME activity is postulated to exist in the apoplast (Hocq et al. 2017). In the guard cells, the unique outward-rectifying K^+^ channel, Guard Cell Outward Rectifying K^+^ channel (GORK), localizes specifically in the polar region (Ache et al. 2000; Eisenach et al. 2014; Jezek and Blatt 2017), implying that it may contribute to the formation of the PME action-favorable microdomain in the polar region through increasing the local apoplastic K^+^ concentration and pH. These data suggests that the spatial regulation of PME activity might control polar stiffening in the guard cells. Further investigations are required to characterize the specific apoplastic microdomain in the polar regions of the guard cells.

### Other potential targets of MYB156 involved in the polar stiffening of the guard cells

**Figure 12.**
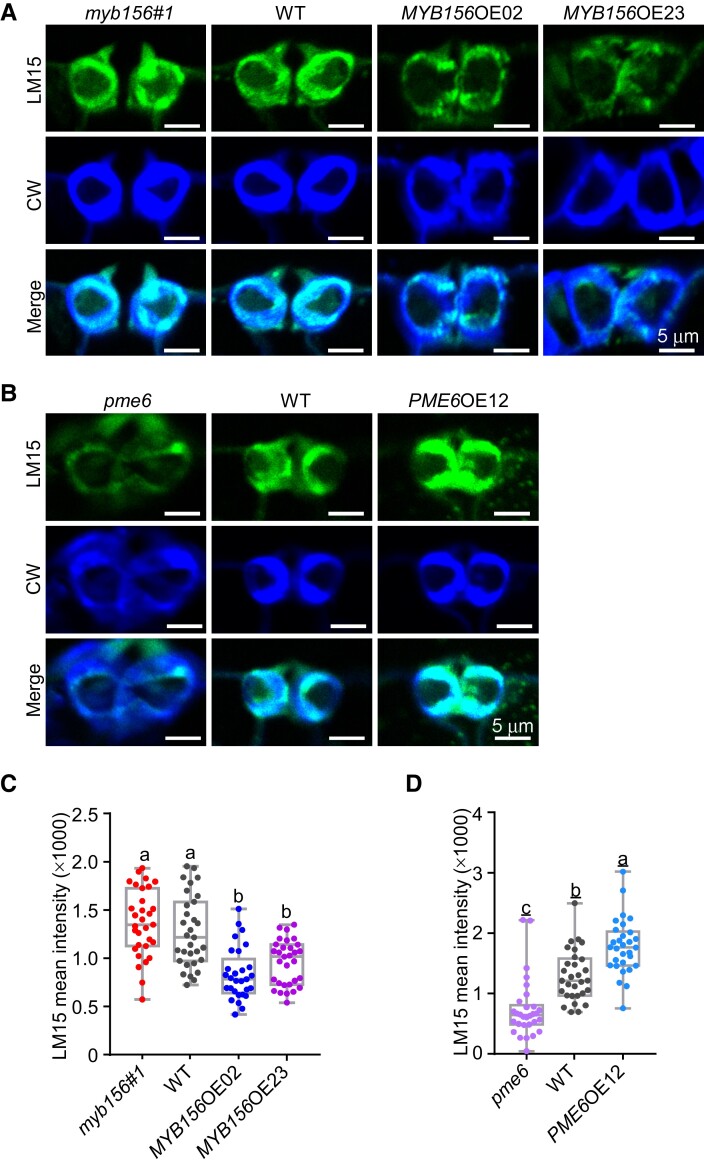
Changes in *MYB156* and/or *PME6* Expression Alter Xyloglucan Abundance of the Guard Cells. **(A)** Representative xyloglucan images of cross-sections of *myb156 #1*, wild-type (WT), *MYB156* overexpression (OE) 02 and 23 guard cells. The cross-section of guard cells from LPI6 leaves (LPI, Leaf Plastochron Index, which was used as an indicator of the leaf age, with LPI6 representing the sixth leaf from the plant top) of 2-month-old plants were immunolabeled with LM15 (in green) and counterstained with CW (in blue) to show guard cell walls. Bar = 5 *μ*m. **(B)** Same as in **(A)**, but labeled in the cross-section of *pme6*, WT, and *PME6*OE guard cells. Bar = 5 *μ*m. **(C)** Quantification of LM15 labeling intensity in cross-sections of *myb156 #1*, WT, *MYB156*OE02 and *MYB156*OE23 guard cells. n = 30 pairs of guard cells from three different plants per genotype. Whiskers extend to min and max, box boundaries represent the 25th percentile (upper) and 75th percentile (lower), the lines inside boxes represent medians, and dots represent the individual data for each pair of guard cells; Different letters indicate statistically significant differences across genotypes, while the same letter indicates no significant difference according to one-way ANOVA Duncan's (D) test (*P* < 0.05). **(D)** Same as in **(C),** respectively, but labeled in the cross-section of *pme6*, WT, and *PME6*OE guard cells.

Although *PME6* acts as a direct target of the transcription repressor MYB156 in stomatal functioning, the loss of *PME6* expression causes less disturbance in stomatal function and polar stiffening than the overexpression of *MYB156* (Figs. 3A, 6, A to E, and 8I), suggesting that there are other downstream targets functioning in the control of the polar stiffening of the guard cells. In this study, we identified several putative *XTHs* from the gene expression profiles of *MYB156*OEs (Fig. 4). XTH is responsible for modifying xyloglucan by cutting and rejoining it, which can loosen the cellulose–xyloglucan network and regulate xyloglucan abundance during plant development (Van Sandt et al. 2007; Zhu et al. 2012; Zhu et al. 2014; Tao et al. 2022). Sufficient xyloglucan production has been shown to be required for proper stomatal function (Rui and Anderson 2016), suggesting that XTH may play a role in MYB156-mediated regulation of the mechanical properties of guard cell walls. To investigate this possibility, LM15 labeling (a monoclonal antibody to xyloglucan) (Marcus et al. 2008) was performed in the genotypes generated in this study. Negative controls for immunolabeling the guard cells of the tested genotypes did not show specific signals (Supplemental Fig. S5). By applying these antibodies/probes, we found that the labeling intensities were unchanged in *myb156* guard cells but significantly decreased in *MYB156*OE guard cells compared to WT guard cells (Fig. 12, A and C). This suggests that *MYB156*OE guard cells contain less xyloglucan than WT guard cells and *myb156* guard cells contain a comparable amount of xyloglucan to WT guard cells. More interestingly, LM15 labeling intensities were significantly higher in *PME6*OE guard cells and significantly lower in *pme6* guard cells than in WT guard cells (Fig. 12, B and D). This suggests that *PME6*OE guard cell walls contain more xyloglucan, and *pme6* guard cell walls, similar to *MYB156*OE guard cell walls, contain less xyloglucan. The observed positive association between demethylesterified HG and xyloglucan content in *PME6*-manipulated transgenic plants (Figs. 6, F, G, J, and K, and 12, B and D) is consistent with the proposed coordinated assembly of pectin and xyloglucan in cell walls through covalent linkage that forms in the Golgi apparatus during xyloglucan biosynthesis (Brett et al. 2005; Popper and Fry 2008).

The association between xyloglucan and pectin in plant cell walls raises the possibility that the detected levels in xyloglucan abundance could be influenced by variations in pectin epitopes and their abundance in *PME6-* or *MYB156*-manipulated lines. This is because previous studies have shown that pectin can mask xyloglucan epitopes within the cell wall, thereby limiting the accessibility of xyloglucan antigen during immunolabeling analysis of cell wall components (Marcus et al. 2008). Nevertheless, *MYB156* may also regulate xyloglucan abundance by manipulating pectin epitopes and their abundance, or by controlling the transcription of genes involved in xyloglucan biosynthesis and modification. Other candidates include PGs and PLLs, both of which function downstream of PMEs, coordinately fulfilling functions in HG modifications (Senechal et al. 2014). The putative ortholog of MYB156 from banana (*Musa acuminata*), MaMYB4, has been shown to directly target the cell wall–modifying genes *MaXTH5* and *MaPG3*, which function in cell wall loosening during fruit ripening (Yang et al. 2022). Further genetic and biochemical studies are required to better understand the detailed contribution of each component to the regulation of polar stiffening in the guard cells.

### Roles of MYB156 in plant development and growth

In this study, we found that *MYB156* is expressed during various stages of stomatal development (Fig. 1C). The fact that MYB156 mediates end-wall thickenings in guard mother cells and polar stiffening in stomata (Fig. 11, C and D) indicates the significance of MYB156 in stoma development. End-wall thickenings are reported to be the symmetric division site of guard mother cells and play important roles in stomatal morphogenesis (Zhao and Sack 1999; Lucas et al. 2006; Zhang and Dong 2018). However, the weakened cell wall polarization did not seem to hinder stoma development in *MYB156*OE plants, as *MYB156*OE plants had similar guard cell index and stomatal patterning, and somehow similar stomatal morphology as WT plants (Fig. 11, C to E and G). These results suggest that *MYB156* perturbation does not disturb stomatal development.

We found that overexpression of *MYB156* limited leaf growth by decreasing HG demethylesterification levels of the cell wall through downregulating the expression of *PME6*. This is consistent with the results of overexpression of rice (*Oryza sativa*) pectin methylesterase inhibitor *PMEI28*, which resulted in a dwarf phenotype (Nguyen et al. 2017). Knocking-out of Arabidopsis *PME3* also impaired root growth (Hewezi et al. 2008). These results imply that decreased demethylesterification of HG corresponds with reduced growth. However, the fact that cell expansion was limited in pavement cells but enhanced in guard cells in *MYB156*OE15 and OE02 lines suggests that the effect of methylesterification level on cell wall rheology and stiffness depends on the context of specific developmental progress in a cell-specific manner. Alternatively, the decreased leaf growth in *MYB156*OE lines may also be attributed to less dynamic stomata. The constant open state of *MYB156*OE stomata results in uncontrolled transpiration, leading to unfavorable plant temperature for CO_2_ assimilation and increased energy waste from excess water absorption and transport. Consequently, cell proliferation and irreversible expansion are limited.

**Figure 13.**
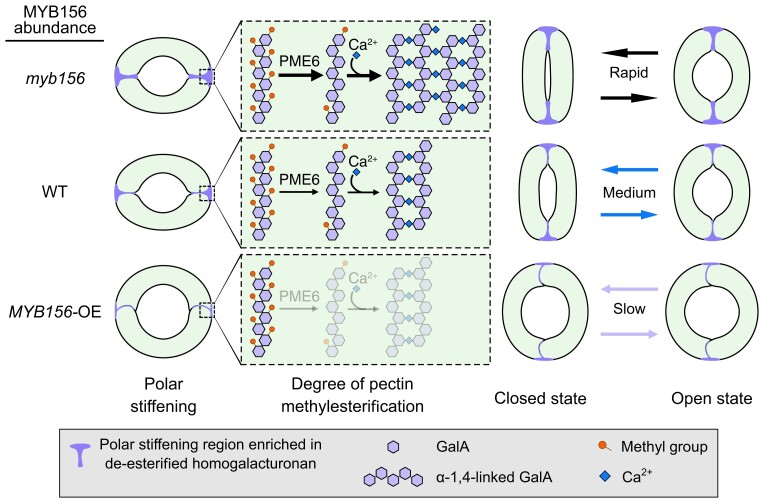
Schematic of MYB156 Regulation of Stomatal Movement. MYB156-mediated polar stiffening through the downregulation of *PME6* in the guard cells is essential for stomatal dynamics and response speed to stimuli, thereby may play a role in improving photosynthesis efficiency and water use efficiency. The knock-out of *MYB156* leads to elevated PMEs activity and more de-methylesterified HG, thereby enhancing the stiffness in the polar regions. As a result, *myb156* stomata not only move rapidly to environmental stimuli but also can close tightly, both of which contribute to water loss reduction under drought stress. In wild-type (WT) plants, the abundance of de-methylesterified HG is maintained at an intermediate level in the polar regions, enabling normal stomatal dynamics. The constitutive expression of *MYB156* in *MYB156* overexpression (OE) plants significantly repressed the levels of PME6, which led to decreases of de-methylesterified HG and wall stiffness in the polar regions. The weakened polar stiffening led to almost constantly opened stomata in *MYB156*OEs. Given that *MYB156* expression was strongly repressed under drought stress, we deduce that the polar stiffening of the guard cells is physiologically regulated in plants for the proper stomatal functioning and environmental adaptation.

The structure–function relationship in guard cells is a long-standing mystery. This study shows that the polar stiffening of the guard cell walls plays an important role in stomatal functioning. The transcription factor MYB156 responds to drought stimuli and acts as a key regulator of stomatal polar stiffening and stomatal functioning, partially through regulating the localized HG methylesterification. Normal *MYB156* expression is required for leaf growth. The polar stiffening process mediated by MYB156 is of great physiological significance for plants to deal with changing environments, specifically because enhanced polar stiffening improves stomatal dynamics and response speed (Fig. 13). Our study provides a promising approach for stomatal engineering through manipulating polar stiffening. The transcription factor MYB156 is a promising target for enhancing plant photosynthetic and water-use efficiency through the production of fast-responding stomata. This can be achieved by specifically reducing its expression in guard cells using guard cell–specific promoters. In future work, using forward genetics to screen for mutants with altered polar stiffening can help identify other molecular players involved in polar stiffening of guard cells.

## Materials and methods

### Plant materials and growth conditions

*P. davidiana* × *P. bolleana* (Shanxin Yang) used in this study was propagated as previously described (Wang et al. 2016). *Populus* clones from 1 to 2-cm lateral buds were cultured on MS medium with 3% (*w*/*v*) sucrose and 0.6% (*w*/*v*) agar in a growth chamber. After 3 wk of growth, the rooted plantlets were transferred into the soil and grown in a climate chamber (25 ± 1 °C, 16 h light/8 h dark cycle, 150 *µ*mol/m^2^/s light [150 W white light LED], 65 ± 5% humidity). For the drought treatment, 120 grams of soil was placed in each pot for cultivating the plants and all *Populus* plants were equally well watered prior to drought treatment. After 2 mo, watering was withheld for the drought-treated plants but maintained for the synchronous control plants. To investigate the effect of high relative air humidity (RH ≥ 85%) on stomatal function and HG methylesterification in the polar regions, rooted plantlets were grown for 2 mo on MS medium in sterile jars under a near-saturated RH. Plants grown in a climate chamber with 65 ± 5% humidity were used as control plants under normal RH.

### Gene expression analysis

For RT-qPCR, RNA was isolated using the RNAprep Pure Kit DP441 (Tiangen) following the manufacturer's instructions. To investigate the effect of drought stress on *MYB156* expression, RNA was isolated from the LPI6 (LPI, which was used as an indicator of the leaf age in this study, with LPI6 representing the 6th leaf from the plant top (Larson and Isebrands 1971)) leaves at various time points (0, 7, 9, 11, 13, and 15 d) after water deprivation. In the *MYB156* expression pattern analysis experiments, RNA was isolated from various tissues of 6-wk-old *Populus* plants. For the analysis of the expression level of genes in the transgenic plants, RNA was isolated from the LPI6 leaves of *Populus* plants. Gene expression with RT-qPCR was conducted as previously described (Zheng et al. 2019) using specific primers (Supplemental Table S1). The relative quantitation results were calculated by normalization to *Populus ACTIN2*. Each experiment was performed with 3 biological replicates, where each replicate consisted of an independent RNA from an individual plant or an independent RNA pool obtained by pooling RNA samples from 3 individual plants.

To visualize the expression pattern of *MYB156* and its target *PME6*, the ∼1.5 kb *MYB156* promoter (*ProMYB156*) and the promoter sequence of *ProPME6* upstream from the ATG start codon of each gene were respectively cloned into the pYBA1121 vector (NCBI:txid1459642) by using restriction enzymes *Kpn* I and *Bam*H I, and pCAMBIA1381 vector (https://www.cambia.org/) by using restriction enzymes *Eco*R I and *Kpn* I. These cloning procedures were performed with specific primers (Supplemental Table S1). The resulting vectors *ProMYB156:GUS* and *ProPME6:GUS* were transformed into *Agrobacterium* (*Agrobacterium tumefaciens*) GV3101 and then transformed into *Populus.* The leaves of transgenic *Populus* were stained for GUS activity as previously described (Zheng et al. 2021).

### Generation of transgenic plants

For constitutively overexpressing *MYB156* and/or *PME6* in *Populus*, the full-length coding region of *MYB156* or *PME6* was amplified from *P. davidiana* × *P. bolleana* using the primers listed in Supplemental Table S1. Then, the *MYB156* was cloned into the pCAMBIA2300 vector (https://www.cambia.org/) using restriction enzymes *Pst* I and *Kpn* I and *PME6* was cloned into the pCAMBIA1307 vector (https://www.cambia.org/) using restriction enzymes *Sal* I and *Xba* I, resulting in *Pro35S* promoter–driven overexpression constructs, *Pro35S:MYB156* and *Pro35S:PME6*. For the construction CRISPR/Cas9 vectors, the target sequences of *MYB156* and *PME6* (Supplemental Table S2) were designed via CRISPR-P 2.0 (http://crispr.hzau.edu.cn/CRISPR2/) and reassembled into sgRNA expression cassettes, following which they were subcloned respectively into a pYLCRISPR/Cas9P_35S_-N and a pYLCRISPR/Cas9P_35S_-H plasmid according to the method reported previously (Ma et al. 2015).

The constructs were introduced into *Agrobacterium* GV3101 and then transformed into *Populus* using the leaf disc method. Leaf explants excised from 3-wk-old plantlets were cut into pieces and then immediately inoculated into the *Agrobacterium* infection solution (Han et al. 2000) for 10 min. Callus induction and shoot and root regeneration were conducted as previously described (Zheng et al. 2021). Transgenic plants for *MYB156* overexpression and gene editing were selected on 50 mg/L kanamycin, and transgenic plants for *PME6* manipulation were selected on 4.5 mg/L hygromycin. Transgenic plants constitutively expressing both *MYB156* and *PME6* were obtained by transforming the *Pro35S:PME6* construct into *MYB156*-overexpressing transgenic *Populus*, *MYB156*OE15, and both hygromycin and kanamycin were used to select transformants during the processes of callus induction and shoot and root regeneration.

The expression levels of *MYB156* and *PME6* in *MYB156* and/or *PME6*-overexpressing plants were determined by RT-qPCR as described above. To analyze the gene editing, the genomic fragment containing the target sequence (300–500 bp) for each gene was amplified using specific primers (Supplemental Table S1) and sequenced after insertion into the pEASY-Blunt Zero vector. The mutated sequences were compared with the WT reference sequence through alignment using the DNAMAN software program (Version 6.0.3.99, Lynnon Biosoft, USA), and the sequencing chromatograms were analyzed using Chromas software (CHROMAS version 2.6.6, www.technelysium.com.au).

### Stomatal function assays

The fully expanded mature leaves (LPI6 and LPI7) from 2-mo-old *Populus* plants were used for stomatal function assays. To investigate stomatal movement, light-induced opening and ABA- or mannitol-induced closing were conducted according to the method reported previously (Ren et al. 2010), with slight modifications. The detached leaves were cut into 1-cm-width strips and floated in opening buffer (10 mM MES-KOH, 10 mM KCl, 50 *μ*M CaCl_2_, 0.1% [*v*/*v*] Triton X-100, pH 6.0) under 150 *μ*mol/m^2^/s light for 2 h to stimulate stomatal opening. The strips were then moved to opening buffer supplemented with 20 μM ABA or 1 M mannitol to induce stomatal closing for 4 h. Stomatal geometries, including stomatal pore width and stomatal complex length in both open and closed states, and pore length and junction length in the open state, were monitored using a Leica DM 5500 B light microscope and measured by ImageJ according to the method reported previously (Yi et al. 2018). The individual plant is used as a replicate for data analysis in stomatal function assay.

To assess stomatal function at the whole-plant level, an LI-6400/XT infrared gas exchange analyzer system was used to monitor stomatal conductance to shifts in CO_2_ conditions under 150 *µ*mol/m^2^/s light and 65 ± 5% relative humidity at 25 °C. The conductance was recorded at changing CO_2_ concentration (from 100 to 1,000 ppm), conducted as described (Amsbury et al. 2016), using a leaf fluorometer chamber (LI-COR) with an LED 2 × 3 Red Blue Light Source. Specifically, the time course of stomatal conductance was examined under ambient CO_2_ conditions (500 ppm) for 30 min, elevated CO_2_ (1,000 ppm) for 50 min, and then reduced CO_2_ (100 ppm) for 45 min. During these periods, the data were recorded every 2 min, except the period of 100 ppm CO_2_ incubation when the data were recorded every 1 min.

To analyze the phenotype of water transpiration through the stomata, water loss measurements were conducted on detached leaves as described previously (Huang et al. 2017). The weights of the detached leaves, incubated under room temperature and room light in laboratory, were measured at various time points (0, 0.5, 1, 1.5, 2, 3, 4, 5.5, and 7 h), with at least 10 replicates (plants). The leaves were oven dried at 80 °C for 48 h and then weighed. The transpiration (water loss) measurement was standardized (%) using the following equation: [(FW_i_ – DW)/(FW_o_ – DW)] × 100, where FW_o_ and FW_i_ are fresh weight at the beginning time point and the following time point, respectively, and DW is the dry weight. As leaf surface temperature is considered a measure of evaporative cooling, which is tightly linked to stomatal function, thermal images of 2-mo-old *Populus* plants were taken using a Testo 890-2 infrared camera in a climate chamber under well-watered or drought treatment conditions. Under drought stress, thermal images of the WT, *myb156 #1*, and *myb156 #2* were taken by an infrared camera at 9 d after water withholding. Images were analyzed with Testo IRSoft, using at least 10 plants.

### RNA sequencing (RNA-seq) and GO enrichment analysis

To identify the target genes of MYB156, RNA-seq was performed in the *MYB156*-overexpressing lines. Total RNA (1 *μ*g) from the LPI6 leaves of 2-mo-old plants was used for library construction with the VAHTS mRNA-seq V3 Library Prep Kit for Illumina. A total of 12 libraries (numbers of libraries = 3 biological replicates × 4 genotypes; for each library, sample was pooled from 4 plants) were sequenced by Illumina NovaSeq 6000 with 150-nt paired-end sequencing. After sequencing, the adapter and low-quality reads were filtered out through Cutadapt (version 1.11) (Martin 2011). Clean reads were mapped to the *P. trichocarpa* reference transcripts by Hisat2 (version 2.1.0) (Kim et al. 2019), allowing up to 2 mismatches. The RSEM (v1.2.6) software was adopted to quantify the transcript abundance based on FPKM (fragments per kilobase of transcript per million fragments mapped) (Li and Dewey 2011). Differentially expressed genes were identified with DESeq2 (Love et al. 2014) with a filter threshold of adjusted *q* < 0.05 and |log2FoldChange| > 1. The raw data were submitted to the NCBI SRA database (https://www.ncbi.nlm.nih.gov/sra) with accession number PRJNA813022. The ClusterProfiler (http://www.bioconductor.org/packages/release/bioc/html/clusterProfiler.html) R package (Yu et al. 2012) was employed to perform GO enrichment analysis (Ashburner et al. 2000). The GO enrichment analysis was calculated using a hypergeometric distribution with a *q*-value cutoff of 0.05. The *q*-values obtained by Fisher's exact test were adjusted with FDR for multiple comparisons.

### Y1H assays

To examine whether MYB156 binds to the *PME6* promoter, the *PME6* promoter fragments (*P1*, TTTCTTTAATTGTTAAGATTCAATTTCTAGAACTAGTGATTTTTTTTTTTTAAT TTTGTTAGGTAGATAGTAATAATTTTAGG, with AC-III *cis*-element underlined; *3AC*, GTTAGGTAGAGTTAGGTAGAGT TAGGTAGA) were synthesized and ligated into the pLacZi2*μ* vector to generate *P1*:*LacZ* and *3AC*:*LacZ* reporter constructs. The MYB156 was fused in frame with GAL4-AD in the pB42AD vector to produce the MYB156-AD activator. Y1H assays were conducted according to the method reported previously (Wang et al. 2019). Yeast (*Saccharomyces cerevisiae*) strain EGY48 cells were cotransformed with the activator and reporter constructs, and transformants were grown on SD/-Trp-Ura plates containing 2% (*w*/*v*) galactose, 1% (*w*/*v*) raffinose, and 40 mg/L X-gal (5-bromo-4-chloro-3-indolyl-β-D-galactopyranoside) for blue color development.

### Electrophoretic mobility shift assay

To examine whether MYB156 directly binds to the *PME6* promoter by EMSA, the coding sequence of *MYB156* was cloned into the pET30a vector for purification of the recombinant protein His-MYB156 from *Escherichia coli*. Meanwhile, the His-tagged protein in the pET30a vector, named His protein here, was also purified and used as a negative control. The 5′-biotin labeled fragment of the *PME6* promoter (harboring the AC motif) and its mutated version (P1^AC^ probe: 5′-TTTGTTAGGTAGATAGTAATAATTTTAGG-3′; and P1^mAC^ probe: 5′-TTTTTTATTTAGATAGTAATAATTTTAGG-3′) were synthesized by Sangon Biotech Co. Ltd. The EMSA was performed using a Light Shift Chemiluminescent EMSA Kit (Thermo) following the manufacturer's protocol. Each 20-*μ*L binding reaction contained 1× binding buffer, 0.05% (*v*/*v*) NP40, 1 mM DTT, 2.5 mM MgCl_2_, and 25 ng/*μ*L Poly (dI•dC). Binding reactions were performed using 200 ng protein and 2.5 ng probe for each of the biotin-labeled promoter fragments at room temperature for 30 min, following which 10 and 50 times unlabeled DNA fragments were added as competitors.

### Transient expression assays

The full-length coding sequences of *MYB156* and ∼1.5-kb promoter of *PME6* were amplified with the primers listed in Supplemental Table S1 and cloned into the binary vectors of pGreenII 62-SK and pGreenII 0800-LUC (Hellens et al. 2005), respectively, producing the *Pro35S* promoter–driven effector vector (*Pro35S:MYB156*) and the *PME6* promoter–driven *LUC* reporter vector (*ProPME6:LUC*). The expression cassette of *Renilla* luciferase (RLuc) was also included in the pGreenII 0800-LUC vector, serving as an internal control. Then, the vectors were introduced into *Agrobacterium* GV3101 and transiently transformed into *Nicotiana benthamiana* leaves by infiltration. Four days later, the LUC activities were detected with the Dual-Luciferase Reporter Assay System (Promega) according to the manufacturer's protocol. Each data point represents 8 reactions based on 8 individual transformed plants in a single experiment. Three independent experiments were performed, all of which produced similar results.

### Immunolabelling and dye staining of guard cell cross-sections

Immunolabelling was conducted following the protocol of Rui *et al*., with the following modifications (Rui et al. 2017). Three-millimeter squares were cut from leaves LPI6 of 2-mo-old *Populus* and fixed in 4% (*w*/*v*) formaldehyde in PEM buffer (0.1 M PIPES, 2 mM EGTA, 1 mM MgSO4, pH 7.0) by vacuum infiltration and then incubated for 1 h. The leaf samples were dehydrated in an ethanol series (1 h each at 30%, 50%, 70%, 100%, ad 100% [*v*/*v*] ethanol) and infiltrated with LR White Resin series (Electron Microscopy Science, Hatfield, PA, USA) diluted in ethanol (1 h each in 10%, 20%, 30%, 50%, 70%, and 90% [*v*/*v*] LR White Resin). The leaf samples were incubated in 100% [*v*/*v*] LR White Resin 3 times, with at least 12 h for each. The leaf samples were then placed in gelatine capsules (Electron Microscopy Science) filled with resin and polymerized at 37°C for a week. Sections of 3-*μ*m thickness were cut by a Fully Automated Rotary Microtome Leica RM2265 (Germany) with a glass knife and placed on Polysine microscope adhesion slides.

Immunolabelling with LM19 (catalog no. LM19, PlantProbes, University of Leeds), LM20 (catalog no. LM20, PlantProbes, University of Leeds), and LM15 (catalog no. AS184203, Agrisera, Sweden) was conducted at room temperature. Sections were blocked with 3% (*w*/*v*) milk protein (BD-Difco Skim Milk) in PBS (137 mM NaCl, 10 mM Na_2_HPO_4_, 2.7 mM KCl, 2 mM KH_2_PO_4_, pH 7.2) for 1 h with gentle rotation. Sections were then incubated with primary antibodies diluted in 3% (*v*/*v*) milk in PBS (1:10 dilution for LM15, 1:50 dilution for LM19 and LM20) for 2 h. Sections were gently washed 3 times with PBS for 5 min each and then incubated with the secondary antibody FITC-conjugated goat anti-Rat IgG (whole molecule) (catalog no. F6258, Sigma) (1:100 dilution in 3% milk [*v*/*v*] in PBS) for 2 h. Samples from this step were kept in the dark, as FITC is light sensitive. Samples were counterstained with a 10-fold dilution of 0.1% (*w*/*v*) Calcofluor White (CW) with PBS for 5 min. Samples were washed 3 times with PBS for 5 min each and then mounted with Citifluor AF1 anti-fade solution (Electron Microscopy Science) on slides. Images were observed with a Nikon inverted fluorescence microscope TE2000-E. The fluorescence of FITC was detected with excitation at 488 nm and emission at 510 nm, and CW with excitation at 405 nm and emission at 455 nm. Negative controls of immunolabeling were conducted with no primary antibody in 3% (*v*/*v*) milk in PBS, and all other steps were the same, including the corresponding parameters during the observation of fluorescence. To quantify the fluorescence signals, the area of the guard cell wall was traced using the CW staining image. The fluorescence mean intensity from the same region was then measured using the NIS-Elements AR Analysis software package, which is designed to work with the Nikon inverted fluorescence microscope TE2000-E. The corresponding negative control was subtracted.

For COS^488^ staining of guard cell cross-sections, sections of 3-*μ*m thickness were incubated with a 1/1,000 dilution of the COS^488^ probe in 50 mM MES pH 5.8 for 5 min and washed 3 times. Quantification of the fluorescence signals was done by measuring the fluorescence mean intensity in the area of the guard cell wall.

### HG labeling of intact guard cells with the COS^488^ probe

The COS probe coupled to Alexa Fluor 488 (COS^488^), received as a gift from Prof. Jozef Mravec (University of Copenhagen, Frederiksberg, Denmark (Mravec et al. 2014)), was used to label de-esterified HG in intact guard cell. Epidermal strips were sampled from fresh LPI6 leaves and submerged into a 1/1,000 dilution of the COS^488^ probe in 50 mM MES pH 5.8 for 20 min. After washing 3 times, the fluorescent signals were observed with a Nikon inverted fluorescence microscope TE2000-E with an excitation wavelength of 488 nm and emission wavelength at 510 nm. The COS^488^ signals around the circumference of the stomatal complex were quantified by Nikon NIS-Elements AR software. For the quantification of COS^488^ signals in the polar regions and outer radial walls, the whole circumference length of the stomatal complex was set to 1 and COS^488^ signals at the relative positions of 0.25 ± 0.03 and 0.75 ± 0.03 were used to indicate the peak signals of the polar regions. Signals at the relative positions of 0.00 ± 0.03 and 0.50 ± 0.03 intervals were used as indicators of the bottom signals of the outer radial walls.

### Atomic force microscopy

For AFM imaging, the LPI6 leaves of 6-wk-old plants were cut into 3 × 3-mm^2^ discs and immersed into 0.55 M mannitol for 1 h. The leaf blocks were fixed onto a glass slide using nail polish and submerged under a drop of 0.55 M mannitol at room temperature while imaging. A silicon nitride cantilever (ScanAsyst-Fluid, Bruker) with a nominal spring constant of 0.7 N/m and a nominal tip radius of 20 nm was used and calibrated by thermal tuning on a glass substrate before each measurement. Young's modulus images were obtained with a commercial AFM BioScope Resolve (Bruker) under PeakForce Quantitative Nanoscale Mechanical (QNM) mode at 64 × 64 pixels with setpoint force 50 nN at 0.5 Hz. The AFM data were processed and analyzed with NanoScope Analysis software (Bruker, version 1.8) and ImageJ software. The stiffness quantification around the stomatal circumference in the polar regions and outer radial walls was conducted as described for COS^488^ signal quantification.

### Plant growth analysis

Two-month-old *Populus* plants were used for plant growth and development analysis. Younger leaves less than 2 cm in length were labeled with COS^488^ to examine the deposition of de-esterified HG during stomatal differentiation. Mature LPI6 leaves were analyzed for fresh weight to monitor plant growth. For analysis of pavement cell size, stomatal density, and index, the LPI6 leaves were excised and soaked in 100 *μ*g/mL PI for 5 min. Images were observed with a Nikon inverted fluorescence microscope TE2000-E. The fluorescence of PI was detected with excitation at 543 nm and emission at 610 nm, using a 10 × 0.45 NA air objective. For guard cell size, the epidermis of a leaf was peeled and monitored using a Leica DM 5500 B light microscope using a 40 × 0.75 NA air objective. Three individual plants with 5 fields each were imaged and analyzed for pavement cell size and stomatal density and index, and 6 individual plants with 5 fields each were imaged and analyzed for guard cell size, using ImageJ.

### Quantification and statistical analysis

Statistical analysis in this study was conducted with Statistical Product and Service Solutions 17.0 (SPSS) (Supplemental Data Set 1). Data are shown as means ± SE. Student's *t*-test and 1-way ANOVA Duncan's (*D*) test were used to analyze statistical significance compared with a control. The *P*-value (*P < 0.05, **P < 0.01) is shown to indicate significant differences. Different letters indicate statistically significant differences across genotypes, while the same letter indicates no significant difference according to 1-way ANOVA Duncan's (*D*) test (*P* < 0.05).

### Accession numbers

The GenBank accession numbers of the *P*. *davidiana* × *P. bolleana* genes investigated in this study are as follows: *MYB156* (OM912833) and *PME6* (OM912834). *P. trichocarpa* Potri gene identifiers for genes referenced in this study are as follows: *MYB156* (Potri.009G134000), *PME6* (Potri.010G109400), *ACTIN2* (Potri.001G309500), *XTH15.1* (Potri.002G236200), *XTH23* (Potri.018G095100), *XTH25.1* (Potri.018G095200), *XTH21* (Potri.018G094900), *XTH25.2* (Potri.013G005700), and *XTH14* (Potri.006G071200).

## Supplementary Material

koad198_Supplementary_Data

## Data Availability

Raw RNA-seq reads are available at the NCBI Sequence Read Archive (https://www.ncbi.nlm.nih.gov/sra) under accession number PRJNA813022. All data needed to evaluate the conclusions in the article are present in the article and/or the Supplemental Information.

## Supplemental data

The following materials are available in the online version of this article.

**Supplemental Figure S1.** Identification of *myb156* knock-out mutants.

**Supplemental Figure S2.** Functional characterization of *PME6*.

**Supplemental Figure S3.** Negative controls for LM19 and LM20 immunolabeling in guard cell walls.

**Supplemental Figure S4.** *MYB156* expression level affects *Populus* leaf size.

**Supplemental Figure S5.** Negative controls for LM15 immunolabeling in guard cell walls.

**Supplemental Table S1.** Primer sequences used in this study.

**Supplemental Table S2.** Target sequence for construction of CRISPR/Cas9 vectors.

**Supplemental Data Set 1**. Statistical analyses.
