# Supplementary material for: The transcription factor MYB156 controls the polar stiffening of guard cell walls in poplar
Source: Plant Cell. 2023 Jul 12;35(10):3757–81. doi: 10.1093/plcell/koad198 (PMC10533337; doi:10.1093/plcell/koad198)
Supplement: koad198_Supplementary_Data [file koad198_supplementary_data.zip › Supplemental Data.pdf]

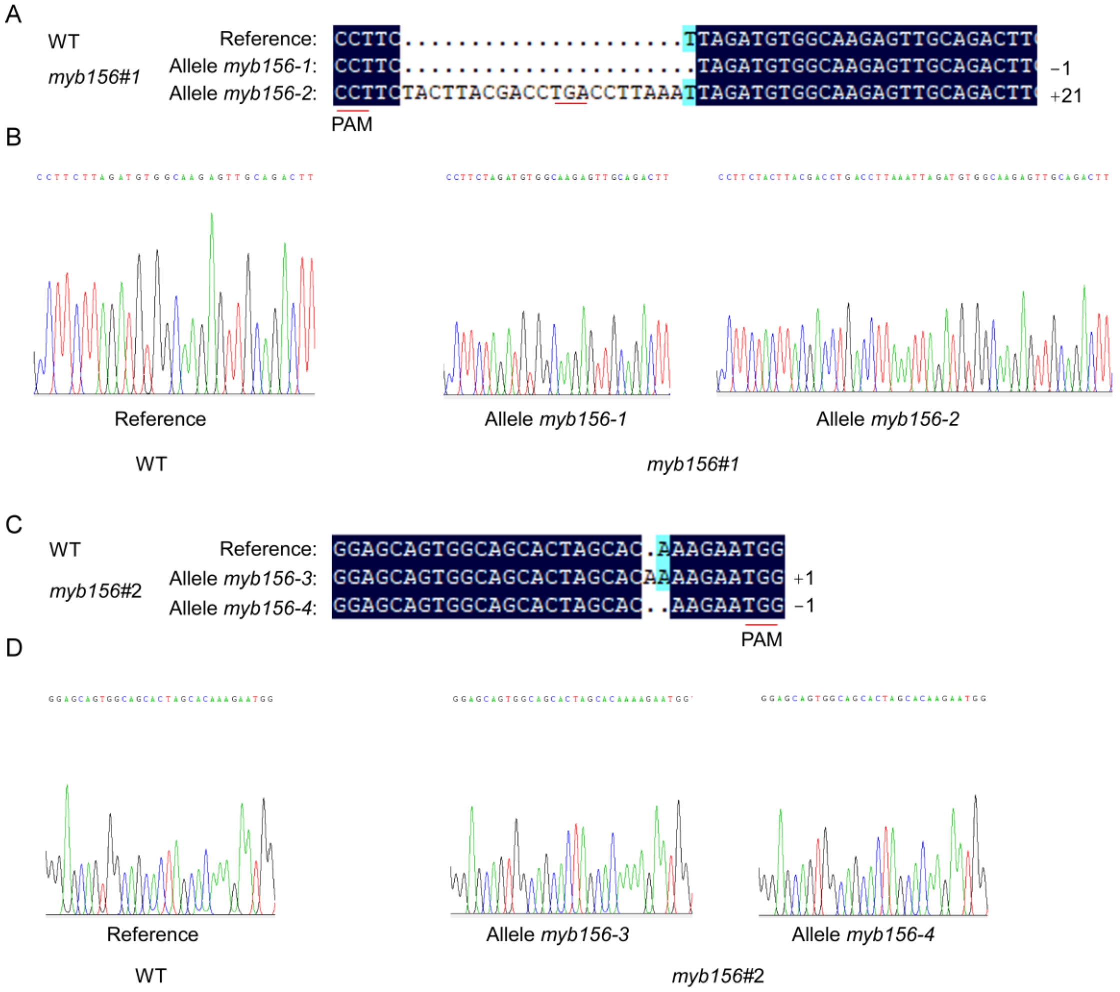

Supplemental Figure S1: Identification of *myb156* Knock-Out Mutants.

(Supports Figure 2).

(A) Confirmation of CRISPR/Cas9-mediated mutation in *myb156#1* by DNA sequencing. Both alleles in the target region contained the same DNA sequence in the wild-type (WT), and one allele is shown. The mutation in *myb156#1* included a 1-bp deletion in allele *myb156-1* and 21-bp insertion in allele *myb156-2*, as shown in the sequence alignment. The PAM sequence (NGG) is underlined in red. The insertion of a 21-bp fragment caused the early termination of the MYB156 protein, and the stop codon (TGA) is underlined in red. **(B)** The sequencing chromatograms of PCR products around the single guide RNA (sgRNA) target regions in WT and *myb156#1*. The PCR products were cloned into the pEASY®-Blunt Zero vector (TransGen Biotech, China). Ten clones were selected for sequencing. **(C)** Confirmation of CRISPR/Cas9-mediated mutation in *myb156#2* by DNA sequencing. The mutation contained a 1-bp deletion in allele *myb156-3* and 1-bp insertion in allele *myb156-4*, as shown in the sequence alignment. **(D)** The sequencing chromatograms of the PCR products around the sgRNA target regions in WT and *myb156#2*. PAM in **(A)** and **(C)** refers to Protospacer Adjacent Motif, a short DNA sequence that is located adjacent to the target DNA sequence and is crucial for the binding and recognition of the Cas9 enzyme. Different colors in **(A)** and **(C)** indicate the level of similarity among the nucleotides at each position within the three alleles. Black indicates that the nucleotides at that position are the same across all three alleles, while blue indicates that only

two alleles share the same nucleotide at that position. In sequencing chromatograms **(B)** and **(D)**, each color represents one of the four nucleotide bases. Green represents adenine (A), red represents thymine (T), blue represents cytosine (C), and black represents guanine (G).

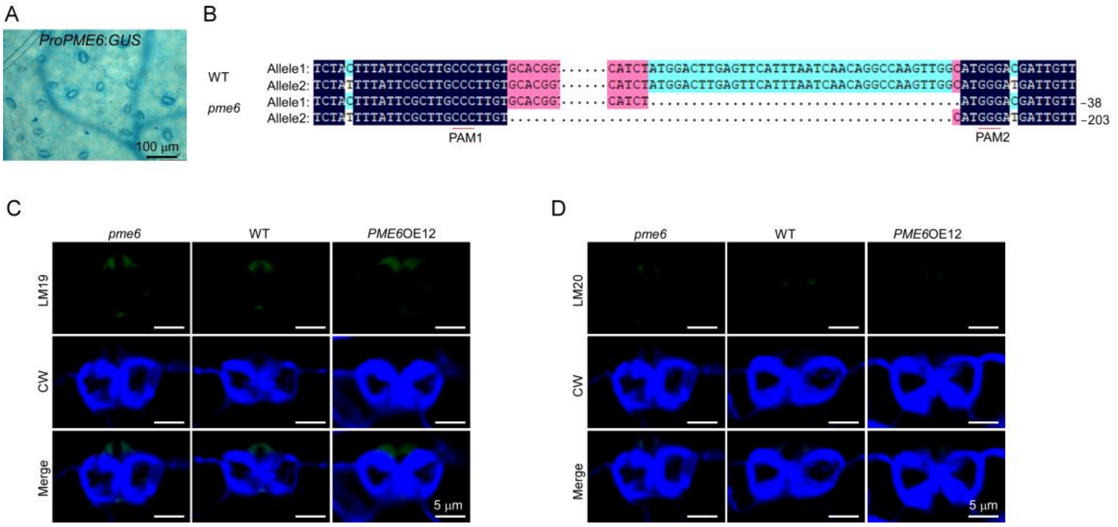

Supplemental Figure S2: Functional Characterization of *PME6*.

(Supports Figure 6).

(A) Expression of *PME6* in guard cells. A representative image of histochemical staining of *Populus* leaves expressing the GUS reporter under *PME6* promoter (*ProPME6*:*GUS*). Strong signals (blue) are shown in the mature guard cells. **(B)** Identification of the *pme6* mutant in *Populus*. CRISPR/Cas9-mediated mutagenesis in *PME6* is shown. The CRISPR/Cas9 construct contains two single guide RNA (sgRNA) target sites, and the PAM sequences (NGG) are underlined in red. The alignment shows a 38-bp and a 203-bp deletion in allele 1 and allele 2, respectively. Different colors show how similar the nucleotides are at each position among the four alleles. The colors go from high to low similarity using a scale of Black, Purple, and Blue. **(C)** and **(D)** Negative controls for LM19 and LM20 immunolabeling in guard cell walls. **(C)** and **(D)** respectively represents the negative control images of Figure 6F and 6H, performed with no primary antibody (as indicated) in each immunolabeling experiment, followed by being counterstained with 0.1% (w/v) Calcofluor White (CW) to trace the guard cell walls (shown in blue channel). *pme6*: *pme6* mutant; WT: wild-type; *PME6*OE12: *PME*6 overexpression 12. PAM: Protospacer Adjacent Motif, a short DNA sequence that is located adjacent to the target DNA sequence and is crucial for the binding and recognition of the Cas9 enzyme. Bars = 5 μm.

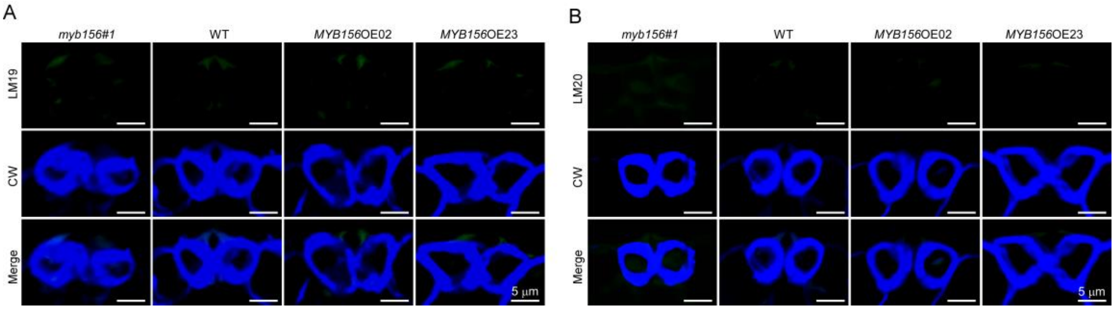

Supplemental Figure S3: Negative Controls for LM19 and LM20 Immunolabeling in Guard Cell Walls.

(Supports Figure 7).

(A) and **(B)** respectively represents the negative control images of Figure 7A and 7C, performed with no primary antibody (as indicated) in each immunolabeling experiment, followed by being counterstained with 0.1% (w/v) Calcofluor White (CW) to trace the guard cell walls (shown in blue channel). *myb156#1*: *myb156* mutant #1; WT: wild-type; *MYB156*OE02 and *MYB156*OE23: *MYB156* overexpression (OE) 02 and 23. Bars = 5 μm.

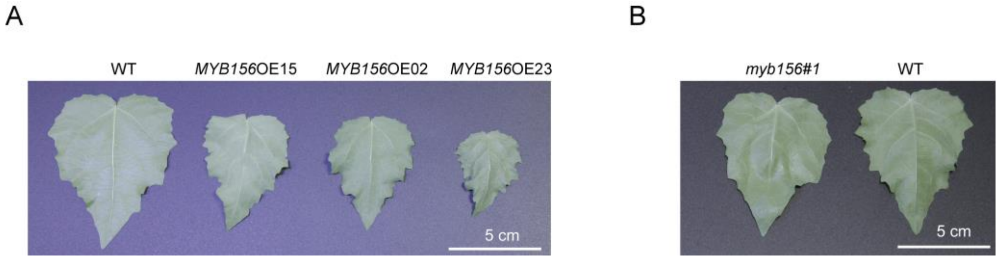

Supplemental Figure S4: *MYB156* Expression Affects *Populus* Leaf Size

(Supports Figure 11).

(A) Representative leaf image of 2-month-old wild-type (WT) and *MYB156* overexpression lines *MYB156*OE15, *MYB156*OE02, and *MYB156*OE23. **(B)** Representative leaf image of 2-month-old WT and *myb156#1*. *myb156#1*: *myb156* mutant #1; WT: wild-type; *MYB156*OE15, *MYB156*OE02, and *MYB156*OE23: *MYB156* overexpression (OE) 15, 02, and 23. Bars = 5 cm.

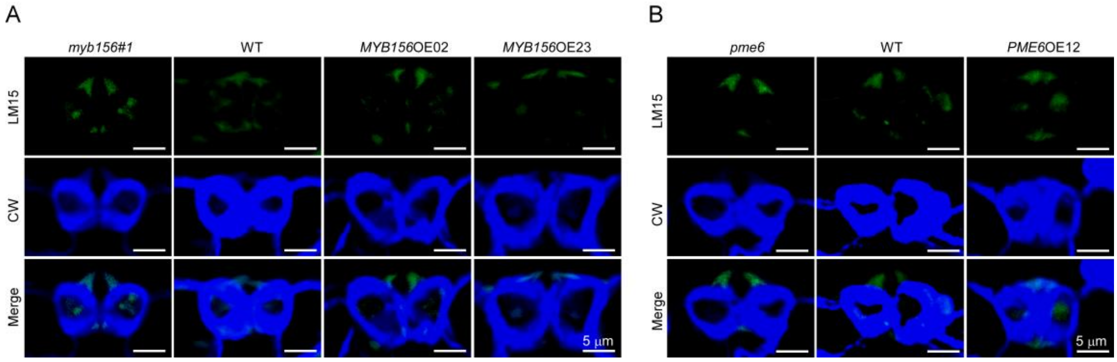

Supplemental Figure S5: Negative Controls for LM15 Immunolabeling in Guard Cell Walls.

(Supports Figure 12).

(A) and **(B)** respectively represents the negative control images of Figure 11A and 11C, performed with no primary antibody in each immunolabeling experiment, followed by being counterstained with 0.1% (w/v) Calcofluor White (CW) to trace the guard cell walls. Green channels represent no primary antibody channel, blue channel represent CW labeling. *myb156#1*: *myb156* mutant #1; WT: wild-type; *MYB156*OE02 and *MYB156*OE23: *MYB156*

overexpression (OE) 02 and 23; *pme6*: *pme6* mutant; *PME6*OE12: *PME*6 overexpression 12. Bars = 5 μm.

Table S1 Primer Sequences Used in This Study

Name	Sequence *	Restriction enzyme
**Primers for overexpression constructs**		
MYB156-F	CTGCAGATGGGAAGGTCTCCATG	*Pst* I
MYB156-R	GGTACCTTTCATCTCTAAACTTCTATAATCC	*Kpn* I
PME6-F	GTCGACATGGATAAGCAGCAGCTTGT	*Sal* I
PME6-R	TCTAGATTATAAACCAGACTCGAAGGG	*Xba* I
**Primers for promoter cloning**		
ProMYB156-F	GGTACCTAAGATTTGAACTGAAACAACATAA	*Kpn* I
ProMYB156-R	GGATCCTTTCATCTCTAAACTTCTATAATCC	*Bam*H I
ProPME6-F	GAATTCTCGGTGCTACCATGTCGG	*Eco*R I
ProPME6-R	GGTACCGGAGACACGAGTTCGGAGG	*Kpn* I
**Primers for mutant identification**		
MYB156-sF	CATCACCAGCCATTCAAGACC	
MYB156-sR	GACCATTTGTTTCCAAGGAGG	
PME6-sF	ATGGATAAGCAGCAGCTTGTTC	
PME6-sR	CGACTTCTTAATCTCCACGTTCT	
**Primers for qRT-PCR**		
ACTIN2-qF	TTCTACAAGTGCTTTGATGGTGAGTTC	
ACTIN2-qR	CTATTCGATACATAGAAGATCAGAATGTTC	
MYB156-qF	AACCAGCCTGATCATCACCA	
MYB156-qR	ACACCACTTTTCATGCCCAA	
PME6-qF	AAAGAACACTGTCACGGCAC	
PME6-qR	TCTCAAGTCTGAAGCTGCCA	
XTH15.1-qF	CAGTAATTGCCAACGCGTCC	
XTH15.1-qR	TGTTTGCTGCGAGTGTTGTG	
XTH23-qF	TTCCTGTGGCACAACCACTT	
XTH23-qR	CATGCTGCATTCCGGAGGTA	
XTH25.1-qF	CAAGAACCTGGAGTCCGTGG	
XTH25.1-qR	ACCAAGCATTCGAGGTGGAG	
XTH21-qF	TAGCTTGCTCACGTACAGCG	
XTH21-qR	CTACTACATGCGAAGAGCGGT	
XTH25.2-qF	TGCTTGTCATTGAGAAAACTGGG	
XTH25.2-qR	GTGGCCAGTTGGACACAGAT	
XTH14-qF	GCAACTAAAGTTGGTGCCCG	
XTH14-qR	AGGTGTGGAAGTCTTTGGTGG	

*Underlined letters represent restriction enzyme recognition sites.

Table S2 Target Sequence for Construction of CRISPR/Cas9 Vectors

Name	Sequence	Promoter
**MYB156**		
Target 1	AGGCAGCACTAGCACAAAGAA	AtU6-29
Target 2	AACTCTTGCCACATCTAAGA	AtU3
**PME6**		
Target 1	ACACTGTCACCATGCAC AA	AtU6-29
Target 2	TCAACAGGCCAAGTTGGCAT	AtU3
