# Supplementary material for: The transcription factor MYB156 controls the polar stiffening of guard cell walls in poplar
Source: Plant Cell. 2023 Jul 12;35(10):3757–81. doi: 10.1093/plcell/koad198 (PMC10533337; doi:10.1093/plcell/koad198)
Supplement: koad198_Supplementary_Data [file tpc22.01028.PeerReviewReport.pdf › tpc22.01028.PeerReviewReport.pdf]

Transcription Factor MYB156 Controls the Polar Stiffening of Guard Cells for Proper Stomatal Functioning in Poplar

Lin Zheng, Yajuan Chen, Liping Ding, Ying Zhou, Shanshan Xue, Biying Li, Jianhua Wei and Hongzhi Wang

Corresponding author: Hongzhi Wang, wanghongzhi@baafs.net.cn, and Jianhua Wei, weijianhua@baafs.net.cn

Review timeline:

TPC2022-RA-00834	Submission received:<br>1st Decision:	Aug. 28, 2022<br>Oct. 18, 2022 *manuscript declined*
**TPC2022-RA-01028**	Submission received:<br>1st Decision:	April 21, 2023<br>June 2, 2023 *accept with minor revisions*
**TPC2022-RA-01028R1**	1st Revision received:<br>2nd Decision:<br>Final acceptance:	June 4, 2023<br>June 14, 2023 *acceptance pending, sent to science editor*<br>July 6, 2023

REPORT: (The report shows the major requests for revision and author responses. Minor comments for revision and miscellaneous correspondence are not included. The original format may not be reflected in this compilation, but the reviewer comments and author responses are not edited, except to correct minor typographical or spelling errors that could be a source of ambiguity.)

TPC2022-RA-00834 1st Editorial decision – *declined* Oct. 18, 2022

Thank you for choosing to send your manuscript entitled "MYB156 controls the polar stiffening of guard cells for proper stomatal functioning in Populus" for consideration at The Plant Cell. Your submission has been evaluated by members of the editorial board as well as expert reviewers in your field, and we regret to inform you that we are not able to recommend the publication of this manuscript in its current form. We have had input from multiple scientists and we have solicited post-review comments.

During the post-review consultation session, we agreed that if you could address the major points raised by the reviewers by new experiments, we would welcome a resubmission. This may be treated as a new submission, but we would attempt to use at least some of the same reviewers. Nevertheless, reviewers may be asked to assess the work as a new manuscript (i.e. are the claims fully supported by the data and do the results presented move the field forward?), and not only whether previous reviewer comments have been addressed.

Specifically, the most important comments in guiding our decision included: (1) a lack of comprehensive cell wall analysis, leaving open the possibility that other wall polymers such as xyloglucan might be more important for influencing stomatal function in Populus; (2) the concern by Reviewer 2 that the work in its current form does not constitute a substantial advance in the understanding of stomatal biomechanics beyond providing further evidence for the polar stiffening hypothesis; and, (3) concerns about the correct treatment and reporting of statistical groups and experimental replicates.

-- Reviewer comments:

TPC2022-RA-01028 Submission received April 21, 2023

Reviewer comments on previously declined manuscript and **author responses:**

Reviewer #1:

Question 1): Throughout the manuscript the authors seem to use the total number of stomata observed as the sample size, n, used in statistical analyses. I am aware that in the stomatal field (past and present) there are various views on what represents a biological-independent observation. I recommend re-working of the statistical analyses of stomatal phenotype using the number of leaves from independent plants as the basis for analysis (lines 629-640: the

number of leaves analyzed is unclear as written). I strongly suspect this will alter the P values shown in, e.g., Fig 2, Fig 3, Fig 6, Fig. 7, Fig 10. It is also advisable (where sensible) to give the precise P value provided by the analysis. If this leads to some of the comparison no longer being "significant" at, e.g., P= 0.05, this may not be a major issue when the data are viewed overall (there is now extensive literature on the danger of chasing P values in statistical analyses. I think that using a better assessment of biological replication than individual stomata (which, theoretically, could all come from a single leaf as the text is written) will strengthen the interpretation of the data.

Responses: According to this comment and along with the concern from Reviewer 3, we reworked the statistical analyses for stomatal function assay using individual plants as a replicate, which was stated in Methods section in the revised manuscript (see lines 662-663). And the statistical information for each experiment is detailed in the corresponding figure legends of Fig. 2, Fig. 3, Fig. 6, and Fig. 10, highlighted in red. Meanwhile, the precise *P* values are also provided in the figures.

Question 2): In Figs 7M and N, it seems that repeated t-tests comparing samples with WT have been made. For 7N where there are 3 comparisons with WT. This might just about be OK, but best practice here (and certainly for Fig 7M with more samples) would be to perform ANOVA followed by some post- hoc test (depending on results from ANOVA).

Responses: In the revised manuscript, the data analysis for Figure 7S and T (respectively corresponding to Figs 7M and N in the original manuscript) was performed by using one-way ANOVA Duncan's (D) test, as suggested. Detailed description of statistical analysis can be found in the figure legend, highlighted in red.

Question 3): I found the labeling of Fig. 5 F and 5G confusing. There seem to be 5 colours (genotypes) above the figures but only 4 lines in Fig. 5G? Clarification and/or better presentation would be helpful.

Responses: Actually, 6 genotypes were investigated in Fig. 5F, and 4 in Fig. 5G. To be clear, each panel was accompanied with its symbol in the revised manuscript rather than shared a common symbol in the original manuscript.

Question 4): How many copies/alleles of *MYB156* are there in *Populus*? I found the description on lines 108-115 unclear. Are there 2 genes or is this 1 paternal/1 maternal allele? Some more introduction to *MYB156* and how the group alighted upon this transcription factor might make this point clearer.

Responses: The *Populus* clone we used in this study is a heterozygous diploid, *Populus davidiana* × *Populus bolleana.* It harbors two alleles of *MYB156*, one from *Populus davidiana* (female parent), the other from *Populus bolleana* (male parent). The biallelic mutant here refers to a plant in which each of the *MYB156* alleles has been altered by CRISPR/Cas9 editing (Here we obtained two biallelic mutants, *myb156*-1 and *myb156*-2). This is in contrast to a monoallelic mutant, which has only one altered or mutated allele. To make this clear, we added some relevant information in the context (see lines 126-132) of the revised manuscript. Meanwhile, we added some introduction to *MYB156* to make this point clearer (see lines 99-100).

<u>Reviewer 2:</u>

Concerns: In this work, the authors hypothesize that transcriptional regulators and signaling cascades might participate in the regulation of stomatal polar stiffening, and suggest that *PME6* is a direct target of MYB156. *PME6* expression was decreased in *MYB156*OEs and increased in *myb156* mutants compared with WT. Manipulation of the expression of *MYB156*/*PME6* affects the pectin methylesterification and the stiffening in the polar regions of guard cells, which in turn impact on stomatal movement speed in the responses to various stimuli. Overall, the work reinforces a notion that pectin methylesterification related polar stiffening has a vital role in stomatal function, and also provides some important genetic evidence, however, my major concern is that the manuscript does not provide sufficient insights into the related field.

Responses: In this study, we found that polar stiffening is the key structure for normal stomatal opening and closing, and is genetically programmed and physiologically modulated in plants. Before this, other researchers have claimed the significance of pectin-based polar stiffening for stomatal opening. They based their claims on two observations: 1) the measurable stiffening at the stomatal poles, and 2) the proper function of young stomata that display a specific nature of polar stiffening (Carter et al., 2017). Compared with their discoveries, we have made essential advances and provided several insights into the field of structure-function relationship of cell walls in

guard cells.

1. **We have identified a specific transcription factor (MYB156) that modulates the specific nature of polar stiffening in guard cells, and dissected the underlying molecular mechanism. Our findings indicate that this specific pattern of cell wall mechanics in guard cells is genetically programmed.**
2. **With the help of genetic approaches, we found that polar stiffening of the stoma is important for both stomatal opening and closing. We provide insight that the role of polar stiffening in guard cell deformation and stomatal movement relies mostly on its role in holding sister guard cells together like cement.**
3. **Most importantly, we first disclosed that the pattern of stiffening at the stomatal poles is responsive to the growth environment. This indicates that polar stiffening is of great physiological significance for plants to deal with the changing environment, and reveals a novel level of dynamics in the guard cell wall.**
4. **Our study provides a promising practical approach for improving stomatal performance through manipulating polar stiffening. This can be achieved by specifically reducing *MYB156* expression in guard cells using guard cell-specific promoters. Additionally, our study suggests using forward genetics to screen for mutants with altered polar stiffening, which can help identify other molecular players involved in polar stiffening of guard cells.**

Question 1.1: Fig 1B, are there any differences with the expression levels of *MYB156* in stomatal lineage cells, if yes, are they coordinated with the pectin modification dynamics if any?

Responses: *MYB156* is expressed during various developmental stages of stomata, including meristemoid, guard mother cell, young stomata, and mature stomata. As a result, the de-methylesterified HG-involved cell wall polarization can be observed before the symmetric division of guard mother cells, suggesting its role in stoma development. To further explain how *MYB156* affects stomatal development, we added new investigations about *ProMYB156*::GUS expression in stomatal lineage cells (see lines 102-104 in Result section, accompanied by Fig. 1C), the HG methylesterification status of guard cell walls during stoma differentiation in wild type and *MYB156*OE plants (see lines 415-419 in Result section, accompanied by Fig. 11C, D), and corresponding discussion (lines 532-543 in Discussion section) to the revised manuscript.

Question 1.2: Fig 1C, the expression of *MYB156* decreases even more dramatically after day 9 under well-watered condition compared with the drought treated plants. What does this mean?"

Responses: The same question was also raised by Reviewer 3. To test whether *MYB156* is developmentally regulated, we compared its expression in leaves LPI 6 of six-week-old, seven-week-old, and eight-week-old plants. We found a dramatic decrease in *MYB156* expression in leaves LPI 6 of eight-week-old plants compared to six-week-old plants (Fig. 1E). This result suggests that *MYB156* is developmentally regulated. We added corresponding explanation in the revised manuscript to clarify this (see lines 105-121). The greater decrease in *MYB156* expression after day 9 in well-watered plants compared to drought-stressed plants may be attributed to well-watered plants being more sensitive and responsive to developmental cues than drought-stressed plants.

Question 2: The two mutant lines generated by CRISPR/Cas9 system, are the two lines used in this study (*myb156*-1 and *myb156*-2) Cas9-free? Whether their defective phenotypes can be rescued by expressing the natural *MYB156* gene?

Responses: The two *Populus* mutant lines (*myb156*-1 and *myb156*-2) used in this study are generated by CRISPR/Cas9 system, but not Cas9-free. It is impossible to obtain Cas9-free, homozygous *Populus* mutant lines through self-pollination of CRISPR/Cas9-edited mutant lines, as can be done in Arabidopsis, for three reasons: 1) *Populus* trees are dioecious; 2) It has a long juvenile period; 3) It generally has a highly complex genetic background, and is reproduced through branch cutting propagation. It is well accepted that biallelic mutants are selected for genetic investigation in *Populus*, in which both the paternal and maternal alleles have been mutated by CRISPR/Cas9 editing at the same time, although they might have different edited versions, like *myb156*-1 and *myb156*-2 mutants used in this study (Supplemental Fig. S1). In this case, the defective phenotypes could not be rescued by expressing the natural *MYB156* gene in the mutant lines, since the introduced *MYB156* gene will be targeted by the CRISPR/Cas9 system in the complementary lines.

Question 3.1: Fig 2B, it seems to my naked eyes that there is no big difference between WT and *myb156*- 2 in terms of the light treatment test results. Is a normal distribution of the data points observed before subjecting them to the statistical significance analysis?

Responses: In the original manuscript, we used the total number of stomata as the sample size in statistical analyses. Based on comments from the reviewers, we reworked the statistical analyses of the stomatal phenotype using the number of plants assayed as the sample size. Changing the biological replication from individual stoma to independent plant has strengthened the interpretation of the data obtained in this study (see Fig. 2B). Meanwhile, the individual data points are also shown in the bar graphs in the revised manuscript. In the case of Fig. 2B, the data distribution is normal (see Fig. 2B).

Question 3.2: Fig 2C, it seems that under ambient 500ppm CO_2, the stomatal conductance of the mutant plants (especially the *myb156*-2) is much smaller than that of WT? Whether there is a possibility that stomatal development is also perturbed?

Responses: As shown at the beginning point of the curve in Fig. 2C, the stomatal conductance of mutant plants is smaller than that of WT under ambient CO_2. Since mutant plants do not show significant difference in growth and plant size to the wild type plants (Fig. 11A, Supplemental Fig. S4B), and their guard cells are normally responsive to various stimuli (Fig. 2A-C), we do believe that stomatal development is not being perturbed in mutant plants.

Question 3.3: Fig 2E, it seems that the size (the biomass) of the drought stressed *myb156*-2 is much bigger than that of the well-watered *myb156*-2? Why?

Responses: In fact, there is no noticeable and statistically significant difference in plant size between the wild-type (WT) and mutant plants, as observed in several replicate drought experiments and shown as fresh weight of mature leaves (Fig. 11A, Supplemental Fig. S4B). Therefore, instead of presenting only one plant in the original version, we have presented three plants in Fig. 2E in the revised manuscript version to better represent the population.

Question 4.1: The OE plants (OE15, OE02 and OE23), is each line with a single T-DNA insertion? According to Fig. 3A and 3D, it seems that there exists a positive correlation between the transcript abundance of *MYB156* and the stomatal conductance under both 500 and 1000ppm CO_2, however other tests for example, the water loss assay deny such a possible scenario.

Responses: *Agrobacterium*-mediated *Populus* transformation often provides transgenic plants with one to a few copies of transgenes. The copy number of transgenes is generally positively correlated with their expression level. In *Populus*, the expression level of transgenes is highly stable over years, and the silencing of transgenes seldom occurs (Hawkins et al., 2003; Li et al., 2009). For these reasons, measuring the copy number of transgenes in *Populus* is unnecessary. Therefore, we did not conduct an experiment to measure the copy number of transgene in *MYB156*OE plants.

The transcript abundance of *MYB156* does show a positive correlation with stomatal conductance under both 500 and 1000ppm CO_2, as a result of differential stomatal stiffening. However, there is no correlation between stomatal conductance and water loss rate (Fig. 3E). This may be due to an undifferential concentration gradient of water vapor from the air spaces of the leaf to the atmosphere among the OE lines. The water loss assay was conducted with detached leaves under normal laboratory conditions, where water from leaves is lost by diffusion of water vapor through the stomatal pore. Water vapor exits the leaf through the stomatal pore after first evaporating into the air spaces of the leaf. Since water moves along this pathway predominantly by diffusion, its movement is controlled by both the concentration gradient of water vapor and the size of the stomatal pore (reflected by stomatal conductance). When the size of the stomatal pore is large enough, the water loss rate is determined by the concentration gradient of water vapor, which should be comparable among the genotypes tested here, leading to no difference in water loss rate for the OE plants. In fact, during the first half hour of the assay, even WT plants have a comparable water loss rate with the OE plants, although their stomatal conductance is much smaller than that of OE plants. As the assay continued, WT stomata closed in response to the stimuli triggered by water loss from leaves, whereas OE stomata did not change much since they lost the ability to close. After the first half hour, WT stomata became small enough to become the limiting factor for water loss, resulting in a much slower water loss rate in WT plants compared to OE plants.

Question 4.2: The enhanced *gs* usually leads to improved plant growth. In Fig3F, under well-watered condition, it seems that WT accumulates more biomass relative to plants of all three lines of *MYB156*OE, whereas in Fig3H, under well-watered conditions, *MYB156*OE15 is much smaller?

Responses: Although enhanced *gs* usually leads to improved plant growth, the *MYB156*OE plants with enhanced gs in this study exhibited a smaller size phenotype compared to WT plants. New investigations demonstrate that the fresh weight of mature leaves was statistically lower in OE lines than in the WT plants (Fig. 11A), consistent with their smaller size. Furthermore, the fresh weight of mature leaves was negatively correlated with gene expression levels among the three *MYB156*OE lines (Fig. 11A and Fig. 3A). Although some plants of *MYB156*OE15 in Fig. 3H appear smaller, *MYB156*OE15 is not the smallest when viewed as a whole population and from a statistical viewpoint. To explain the roles of MYB156 in plant growth, we added a new subsection in Result section (see lines 393-430, accompanied by Fig. 11 and supplemental Fig. S4), a corresponding part in Discussion section (see lines 532-559) and Method section (see lines 829-841).

Question 4.3: In addition, it seems that the leaf color of OE plants is much darker that of WT, it this true? If yes, the possible biological relevance of this phenotype?

Responses: Yes, the leaf color of *MYB156*OE plants is indeed darker than that of WT, indicating that chlorophyll synthesis is enhanced in *MYB156*OE plants. Further investigation is needed to determine how the status of extracellular pectin and cell wall stiffness is involved in the synthesis of chlorophyll in *MYB156*OE plants.

Question 5: The transcript levels of *PME6* in *PME6*OE12 and *PME6*OE13 are significantly different (Fig. 5E), why no significant difference to *gs* was observed in *PME6*OE12 and *PME6*OE13 (Fig.5G)? I think developing and phenotyping the *pme6myb156* double mutant and the transgenic plants that overexpress *MYB156* in the background of *pme6* mutant will be informative, if MYB156 acts (partially) through negatively regulating the expression of *PME6*.

Responses: The difference in *gs* between *PME6*OE12 and *PME6*OE13 is unknown because *gs* measurements were not conducted on *PME6*OE13 (Fig. 5G). However, a significant difference in water loss rate was observed between *PME6*OE12 and *PME6*OE13 (Fig. 5F), suggesting that the expression level of *PME6* has a corresponding effect on stomatal function.

Developing and phenotyping the *pme6myb156* double mutant is a great suggestion. We introduced the Cas9/*PME6*-sgRNA vector into *myb156*-1 mutant and obtained hundreds of transgenic lines. Unfortunately, we only identified a few monoallelic mutations in *PME6*. In *Populus* genetic research, Cas9-mediated mutants must be identified as biallelic mutants at the T0 generation. This is because monoallelic mutants cannot be used to obtain biallelic mutations through self-pollination, as is done in *Arabidopsis*, due to the nature of dioecism and the long juvenile period of 5-8 years. The rarity of biallelic mutations compared to monoallelic mutations in Cas9-mediated *Populus* mutants, combined with the time-consuming *Populus* transformation procedure and clonal propagation from a single transgenic seedling to obtain a population for several replicates of physiological experiments, makes the generation and phenotyping of mutants difficult. Despite this, based on our experience, hundreds of transgenic plants are enough to screen out assured loss-of-function mutations in both alleles in the first round of transformation. We have no idea why the mutation rate of the target gene was much lower in the second round of transformation than in the first. Concerns arise when developing transgenic plants that overexpress *MYB156* in the background of the *pme6* mutant. Differential expression levels of *MYB156* may complicate the phenotyping of the transgenic plants when compared to the *MYB156*OE lines. In *Arabidopsis*, researchers can manipulate multiple genes by cross-breeding transgenic lines, which allows for comparable expression levels. However, manipulation of multiple genes can be conducted only via multiple transformation in *Populus*, which is very challenging. Despite this, we attempted to mutate *PME6* in the background of one of the *myb156*OE lines. Unfortunately, we have not yet screened out any assured loss-of-function mutation in both alleles.

Regardless of whether the aforementioned transgenic lines were obtained or not, the experiments presented in this manuscript have included the generation and phenotyping of a range of transgenic plants (both overexpressing and gene-edited, and those that simultaneously overexpress two genes from a postulate pathway). These analyses were combined with immunolabeling, transcriptomics, and molecular biology, among other things. Both genetic and molecular studies have demonstrated that MYB156 plays a role in stomatal functioning by negatively regulating the expression of *PME6*.The result that *PME6* knocking-out causes less disturbance in stomatal function and polar stiffening than the overexpression of *MYB156* implies that MYB156 acts in stomatal functioning partially through negatively regulating the expression of *PME6*. Therefore, we believe that the conclusions in this manuscript are

solid and convincing.

Reviewer 3:

Question 1: Stomatal aperture measurements (Fig2): The authors report n=>100 here, however adjacent stomatal complexes on the same leaf are not independent replicates as stomata respond in patches, for statistical purposes here n should be the number of plants assayed

Responses: This is also the concern of other reviewer. As suggested, we have reworked the statistical analyses for stomatal function assay using individual plants as a replicate, which was stated in Methods section in the revised manuscript (see lines 662-663). And the statistical information for each experiment is detailed in the corresponding figure legends of Fig. 2, Fig. 3, Fig. 6, and Fig. 10, highlighted in red. Meanwhile, the precise *P* values are also provided in the figures.

Question 2: Towards the latter end of the paper is where my major issues is. Namely that the authors have only looked very superficially at cell wall composition. This transcription factor appears likely to bind to multiple cell wall components - so it's effects may well be additive across different polysaccharides. By only using cos488 it makes it hard to conclude that changes observed are due to esterification status of pectin, it could also be due to changes in overall pectin abundance, or XyG (which has also been shown to impact stomatal dynamics). Specifically in some stomata the complex seems completely detached, this is quite an extreme phenotype suggesting a fundamental loss of adhesion, more reminiscent of pectin biosynthesis mutants (albeit in a very localised manner). Using antibodies such as LM19/20 or JIM7 would have enabled a more complete view of pectin abundance. This can be addressed in two ways - a) Additional experimental data to broaden out the cell wall analysis - probably including xyloglucan in addition to different pectin epitopes. OR b) Revise the discussion and results to reflect these uncertainties.

Responses: We probed the guard cell cross-sections with the suggested antibodies (LM19/20 and LM15) to comprehensively analyze the composition of guard cell wall in genotypes generated in this study. Our new experiments imply that MYB156 might regulate xyloglucan abundance in guard cell walls through manipulating the abundance of de-methylesterified HG. The new analysis of the cell wall composition in guard cell cross-sections led to revisions in Result section (see lines 237-278, lines 283-299, lines 303- 310), in Discussion section (see lines 496-523), and in Method section (see lines 755-796). Additionally, based on data in reference papers, we still suggests that abundance of de-esterified HG, rather than HG, play a more important role in cell adhesion (see lines 454-462).

Minor Points:

Question 1: The authors describe pectins, but are really only referring to HGA which is a subclass of pectin - would be ideal to clarify this somewhere to ensure clarity.

Responses: Yes, the pectin here is referring to homogalacturonan (HG). So we changed it into pectic homogalacturonan or HG in the context. All changes are highlighted in red.

Question 2: line 69: I would dispute that pectin is the major regulator - many other components have large impacts on mechanical properties - perhaps change to "one of the major regulators"

Responses: We agree with this comment and have made the modification in the manuscript (see line 72).

Question 3: Figure 1: This data is clear except it is not clear to me why *MYB156* decreases even under well-watered conditions - is there some temporal/developmental regulation of this? I would like the authors to clarify this either in their response to reviews (or ideally in the manuscript)

Responses: Our new experiment suggests that *MYB156* is developmentally regulated (Fig. 1E). We added corresponding explanation in the revised manuscript (see lines 105-121, accompanied by Fig. 1E).

Question 4: The genetics in this article are very good, multiple lines used and appropriate controls. In particular in Fig3: The authors achieve good level of overexpression, and the effects seem to be driven by expression (lowest OE line also has lowest functional effect) - this supports the authors conclusions well

Responses: Thanks for such good encouragement.

Question 5: I am not clear why the authors did not do stomatal aperture measurements in fig3 for the overexpressors - this is not essential but if the authors have would be a nice panel to include.

Responses: To our knowledge, the term of "stomatal pore width" in Fig. 3 and other Figures that present stomatal phenotypes indeed refer to "stomatal aperture".

Question 6: Figure 6: PME6 has been shown to impact more than just the polar regions in *Arabidopsis*, I think to strongly conclude that there is no difference in the outer radial walls then transverse sections would also be needed - perhaps tone down this statement.

Responses: COS^{488} probing on guard cell transverse sections showed that overall de-methylesterified HG was decreased in *pme6* guard cell (Fig. 6J, K). So we have toned down this statement (see lines 256-258).

Notes/future directions:

Question 7: Transcription factor regulation of CW a very nice finding - would love to see this exctopically expressed under inducible/tissue specific promoters to see if CW properties can be artificially modulated in a controlled way (for the future - not needed for this paper)

Response: This is a great idea, and guard cell-specific/drought-induced promoter is worth trying.

Question 8: Would be interesting to see if this also modulates stomatal Xyg

Responses: Through antibody LM15 immunolabeling, it was found that Xyg is also modulated in guard cells. The results show that manipulating both *MYB156* and *PME6* alters xyloglucan abundance in guard cell walls. This implies that *MYB156* may regulate xyloglucan abundance in guard cell walls by either regulating the transcription of genes involved in xyloglucan synthesis and modification or manipulating the abundance of de-methylesterified HG (see lines 496-523).

References:

Carter R, Woolfenden H, Baillie A, Amsbury S, Carroll S, Healicon E, Sovatzoglou S, Braybrook S, Gray JE, Hobbs J, Morris RJ, Fleming AJ (2017) Stomatal opening involves polar, not radial, stiffening of guard cells. Curr. Biol. 27: 2974-2983 e2972

Hawkins S, Leple J-C, Cornu D, Jouanin L, Pilate G (2003) Stability of transgene expression in poplar: A model forest tree species. Annals of Forest Science 60: 427-438

Li J, Brunner AM, Meilan R, Strauss SH (2009) Stability of transgenes in trees: expression of two reporter genes in poplar over three field seasons. Tree Physiol. 29: 299-312

TPC2022-RA-01028 1st Editorial decision – *accept with minor revisions* **June 2, 2023**

We have received reviews of your manuscript entitled "Transcription factor MYB156 controls the polar stiffening of guard cells for proper stomatal functioning in poplar." On the basis of the advice received, the board of reviewing editors would like to accept your manuscript for publication in The Plant Cell. This acceptance is contingent on revision based on the comments of our reviewers. In particular, please consider the following:

If you agree with the presence of the uncertainty highlighted by Reviewer #3, please update the text to reflect this uncertainty.

-- Reviewer comments:

TPC2022-RA-01028R1 1st Revision received **June 4, 2023**

Reviewer comments on previous submission and **author responses**:

Reviewer #1:

The authors have successfully addressed the points raised in my original review.

Although not directly related to my initial review, I note that as a result of further cell wall analysis in the new manuscript it appears that changes in xyloglucan levels also occur in a number of the lines examined. This is a very interesting observation but obviously complicates the interpretation of the pectin-related data. The authors have added the xyloglucan points to the Discussion and the possibilities raised are reasonable, but clearly the story is not as simple as in the original version of the manuscript. However, I think the main conclusions on polar stiffening are still valid.

Responses: We sincerely appreciate the reviewer's positive feedback regarding the revisions made to our manuscript. In response to the new comments from reviewer #3, we have once again made slight modifications to the xyloglucan section, as indicated in lines 515, 519-525, and 527. We want to assure the reviewer that these modifications will not impact the main conclusions drawn regarding polar stiffening.

Reviewer 2:

The authors have addressed my concerns very well. I only have one comment about the requirement of MYB156 for leaf growth in poplar. I don't think the data provide much insight into how the leaf growth is regulated, or the results are really useful to advance our understanding of the stomatal regulation.

Responses: We appreciate the reviewer's positive feedback on our manuscript revisions. Regarding the concern raised about the requirement of MYB156 for leaf growth, we would like to clarify the purpose of including the data on transgenic plant growth in our study. The intention was to provide a comprehensive and unbiased presentation of our findings. While we agree that the data may not directly elucidate the regulatory mechanisms underlying leaf growth or significantly advance our understanding of stomatal regulation, they do serve to demonstrate that *MYB156* perturbation does not interfere with the identification and development of stomata. By observing the limited growth of the *MYB156* overexpression lines, we can infer that the defective stomatal dynamics, at least partially, contribute to the reduced leaf growth. This observation further highlights the significance of polar stiffening in stomatal functioning and plant physiology.

Reviewer 3:

I am pleased to see the updated version of this article. The authors have made a number of changes which I believe improves the original manuscript and makes the data clearer. In particular I am pleased that the authors have modified their discussion of cell walls to tone down some of their conclusions. I still have some questions and comments about the paper, but overall I feel the authors have made a good effort to answer all my questions.

My one remaining concern is in the cell wall compositional analysis, as proposed in my original review the authors now show data suggesting that XyG has also changed in the cell wall, although not in the polar manner shown for HG, this suggests that the transcription factor may controls multiple cell wall modifying genes from multiple synthetic pathways - although it is impossible to know which effects are direct, and which are indirect.

I am slightly puzzled as to why the authors decided to use LM15 to probe XyG rather than the more popular LM25, which accommodates a broader range of substitutions. I am wary of the authors conclusions regarding the XyG labelling. It has been previously shown that pectin can mask XyG by spatially restricting antigen-antibody interactions (https://bmcplantbiol.biomedcentral.com/articles/10.1186/1471-2229-8-60) , the authors should have enzymatically removed the pectin, to reveal the underlying XyG, as it stands this evidence does not identify whether XyG abundance is changed, or whether the changes to pectin have simply changed XyG antigen accessibility. I do not believe this distinction is crucial for the paper, as the XyG is not the focus - rather this is to point out to readers that the TF may modify more components than just pectin. However, I do believe that the authors should modify the text to reflect this uncertainty.

Other than the above concern I am satisfied that my other comments, which mostly related to the statistical analysis or toning down of statements in the text have been satisfactorily addressed and that these changes strengthen the manuscript compared to its original version.

As an addendum I think that identification of cell wall modifying transcription factors will, in coming years prove to be a crucial tool for the cell wall research community in trying to unpick cell wall structure-function relationships.

Responses: We are pleased to hear that the changes we made have improved the clarity and overall quality of the

paper, and appreciate the acknowledgement that most of the reviewer's previous comments and concerns have been satisfactorily addressed. Regarding the new concern about using LM15 instead of the more popular LM25 to probe XyG, we would like to clarify that at the time of our study, LM25 was not available to us. Consequently, we chose to use LM15 as an alternative method for detecting XyG. In our pre-experiments, we applied pectate lyase to the wild-type sections and found that it had no discernible effect on LM15 labeling intensity compared to the control without enzyme treatment. Based on this observation, we decided not to include enzymatic treatment in the LM15 labeling analysis. However, upon revisiting the reference paper, which also mentioned by the reviewer, and reconsidering our conclusions, we agree with the uncertainty surrounding the analysis of XyG abundance. Had we used an invalidating enzyme during the pre-experiment, it might have affected the detection of XyG. In order to ensure the most objective interpretation of our study's results, we have incorporated the acknowledgement of the uncertainty into the revised manuscript (see lines 515, 519-525, 527).

TPC2022-RA-01028R1 2nd Editorial decision – *acceptance pending* **June 14, 2023**

We are pleased to inform you that your paper entitled "Transcription Factor MYB156 Controls the Polar Stiffening of Guard Cells for Proper Stomatal Functioning in Poplar" has been accepted for publication in The Plant Cell, pending a final editorial review by a science editor.

Final acceptance from Science Editor **July 6, 2023**
